# B Cells and Ectopic Follicular Structures: Novel Players in Anti-Tumor Programming with Prognostic Power for Patients with Metastatic Colorectal Cancer

**DOI:** 10.1371/journal.pone.0099008

**Published:** 2014-06-06

**Authors:** Anastasia Meshcheryakova, Dietmar Tamandl, Erika Bajna, Judith Stift, Martina Mittlboeck, Martin Svoboda, Denise Heiden, Stefan Stremitzer, Erika Jensen-Jarolim, Thomas Grünberger, Michael Bergmann, Diana Mechtcheriakova

**Affiliations:** 1 Department of Pathophysiology and Allergy Research, Center of Pathophysiology, Infectiology and Immunology, Medical University of Vienna, Vienna, Austria; 2 Department of Surgery, Medical University of Vienna, Vienna, Austria; 3 Department of Pathology, Medical University of Vienna, Vienna, Austria; 4 Center for Medical Statistics, Informatics, and Intelligent Systems, Medical University of Vienna, Vienna, Austria; 5 Comparative Medicine, Messerli Research Institute of the Medical University of Vienna, Veterinary University of Vienna and University of Vienna, Vienna, Austria; Institut Pasteur, France

## Abstract

Remarkably limited information is available about biological mechanisms that determine the disease entity of metastatic colorectal cancer in the liver (CRCLM) with no good clinical parameters to estimate prognosis. For the last few years, understanding the relationship between tumor characteristics and local immune response has gained increasing attention. Given the multifaceted roles of B-cell-driven responses, we aimed to elucidate the immunological imprint of B lymphocytes at the metastatic site, the interrelation with macrophages, and their prognostic relevance. Here we present novel algorithm allowing to assess a link between the local patient-specific immunological capacity and clinical outcome. The microscopy-based imaging platform was used for automated scanning of large-scale tissue sections and subsequent qualitative and quantitative analyses of immune cell subtypes using lineage markers and single-cell recognition strategy. Results indicate massive infiltration of CD45-positive leukocytes confined to the metastatic border. We report for the first time the accumulation of CD20-positive B lymphocytes at the tumor – liver interface comprising the major population within the large CD45-positive aggregates. Strikingly, functionally active, activation-induced cytidine deaminase (AID)-positive ectopic lymphoid structures were found to be assembled within the metastatic margin. Furthermore, the CD20-based data set revealed a strong prognostic power: patients with high CD20 content and/or ectopic follicles had significantly lower risk for disease recurrence as revealed by univariate analysis (p<0.001 for both) and in models adjusted for clinicopathological variables (p<0.001 and p = 0.01, respectively), and showed prolonged overall survival. In contrast, CD68 staining-derived data set did not show an association with clinical outcome. Taken together, we nominate the magnitude of B lymphocytes, including those organized in ectopic follicles, as novel prognostic marker which is superior to clinicopathological parameters. Findings emphasize anti-tumoral role of B cell-driven mechanism(s) and thus indicate a new way of thinking about potential treatment strategies for CRCLM patients.

## Introduction

Metastatic colorectal cancer remains a therapeutic challenge despite recent improvements in systemic treatment including developments in cytotoxic and targeted agents [1], [2] and multidisciplinary management. Remarkably limited information is available regarding biological mechanisms that determine the prognosis of this disease and could be considered as entry points for novel therapeutic interventions. Furthermore, there are no clinical parameters available to estimate survival prognosis of patients after liver resection. However, there is evidence to consider colorectal cancer liver metastases (CRCLM) as an immunogenic cancer type since increased density of T lymphocytes at the metastatic margin with anti-tumoral T-cell effectors-driven mechanisms was shown to be prognostically important [3].

It is generally accepted that solid tumors represent an example of tissue where immune cells, either resident or infiltrating, might get aberrantly activated thereby affecting the clinical course of disease [4], [5]. However, solid tumors of different origin can vary considerably in leukocyte composition. Given contribution of either type of immune cells – cytotoxic T lymphocytes (CTLs), T helper (Th) cells, regulatory T (Treg) cells, NK cells, DCs, monocyte/macrophage lineage cells, and B-cell subsets – the tissue-specific mechanisms for each respective tumor type linking patient-specific immunological capacity and the clinical outcome might be considered. To describe best the cancer type-attributed and patient-orientated heterogeneity in amount and/or types of infiltrating immune cells and their distribution patterns within the tumor as well as the consequent stepwise events to generate tumor-accompanying immune response, a new terminology was integrated such as *the immune landscape*, *the immune contexture, the Immunoscore, the immunome*, and *the cancer-immunity cycle* [4], [6]–[8]. On the one hand, solid tumors are generally characterized as immunosuppressive based on multiple networking mechanisms including activation of immunosuppressive pathways and signals, induction of tolerance to tumor antigens and recruitment of immunosuppressive cells such as Treg cells, myeloid-derived suppressor cells, and tumor-associated macrophages (TAMs) [9]–[11]. On the other hand, studies focusing on T cells proposed a strong anti-tumoral potential of CD8-positive CTLs in patients with various cancer types [12]. Noteworthy, there is strong positive association between density of infiltrates with cytotoxic and memory T-cell phenotypes and prolonged patient survival with primary and metastatic colorectal cancer [3], [13]–[16].

Less is known about the functional role of tumor/stroma infiltrating B cells regarding the mechanisms underlying pro- versus anti-tumor programming of neoplastic tissues. The variety of B-cell-mediated actions includes the antigen-dependent T-cell priming, the machinery for the generation of immunoglobulins, secretion of cytokines and chemokines that mediate (promote or inhibit) cell differentiation and inflammation [17] thus contributing to disease pathogenesis through antibody-dependent and -independent mechanisms [18]–[22].

Additional relatively new aspect linking B cell biology and inflammation is based on the discovery of germinal center (GC)-like structures outside of secondary lymphoid organs. Indeed, multiple studies have demonstrated the existence of ectopic follicular structures (also called tertiary lymphoid structures) at sites of chronic inflammation within different tissues, suggesting class switching of immunoglobulins and somatic hypermutation events to take place locally under the control of activation-induced cytidine deaminase (AID) ([23]–[26], reviewed in [13], [27], [28]). Importantly, apart from benign tissues with chronic inflammatory processes, ectopic follicular structures have been reported in some types of solid tumors. The presence of tertiary lymphoid structures were studied in non-small-cell lung cancer patients and found to be associated with favourable clinical outcome; either density of mature DCs [29] or, additionally, density of follicular B cells [30] were used to assess the prognostic effect of tertiary lymphoid structures. Ectopic follicular structures were also detected in patients with infiltrating ductal carcinoma of the breast, suggesting an *in situ* antigen-driven B-cell response to a variety of tumor- and breast-associated antigens [31]. In respect of primary colorectal cancer (CRC), in a pilot study, ectopic follicular structures were predicted by immune gene array profiling and confirmed by immunostaining [32].

Given the evidence supporting the potential contribution of tumor infiltrating B cells to the disease pathogenesis, progression and/or disease resolution, we aimed to investigate the B-cell-driven immune control in a patient cohort with colorectal cancer liver metastases (CRCLM). Yet, a general weak point for evaluation the presence and prognostic effect of resident and/or infiltrating immune cells across the large-scale patient specimen with CRCLM (or other type of complex malignant tissue) is the requirement for quantification algorithms. Indeed, the need for automated, reproducible quantification of immune cell populations based on histological evaluations has been emphasized by the scientific community within translational research area and certain algorithms have been described [33]–[35]. The most successful example of immune scoring assessment is based on the ratio of T-cell subsets (CD3^+^, CD8^+^, CD45RO^+^, and Granzyme B^+^) in patients with primary colorectal cancer. Immunoscore was proposed as a prognostic marker to be used in routine testing [36], [37]; to promote the worldwide use of Immunoscore the task force was initiated among 22 international expert centres [38].

In the current study, we used an automated TissueFAXS-based microscopy system and a newly established algorithm for detection and quantification of immune cell populations across a large-scale specimen of CRCLM. Given the multifaceted roles of B-cell-driven responses particularly at sites with chronic inflammation, we aimed to elucidate the patient-specific imprint of B lymphocytes at the metastatic border with particular focus attributed to the phenomenon of ectopic follicle formation, interconnection between B cells and infiltrating/resident macrophages, and assessment of their prognostic effect.

## Material and Methods

### Ethics statement

The study was approved by the Ethics Committee of the Medical University of Vienna (EK-Nr. 1277/2012). The informed consent was waived by the institutional review board due to the retrospective nature of the study.

### Profile of study patients

A panel of paraffin-embedded specimens of patients with CRCLM was retrieved retrospectively from 65 patients that underwent surgery at the Department of Surgery, Medical University of Vienna. First group consists of 14 patients who had a liver resection between 2001 and 2007, had metachronous presentation of metastatic disease (no metastases when the primary tumor was diagnosed) and underwent resection without preoperative chemotherapy (panel I). The median follow up time for this group was 50.2 months (95% CI: 33.6–66.8 months). This cohort of patients allowed us to assess the immunological imprint in treatment-naïve specimens. Second group (panel II, n = 51) included randomly chosen patients who received chemotherapy prior to liver resection; inclusion date was between 2006 and 2009; the median follow up time was 32.2 months (95% CI: 24.4–40.0 months). As a result of random selection, the panel II included those patients who had fluoropyrimidine-based cytotoxic chemotherapy in combination with oxaliplatin (46 patients) or with irinotecan (5 patients). Additionally, all patients received bevacizumab as the preferred regimen at our institution during this time period. Patients received their last chemotherapy treatment 21 days before surgery; the last bevacizumab treatment was given up to 5 weeks before surgery. Within panel II, 19 out of 51 specimens were evaluated for CD45, while all 51 specimens were evaluated for B-cell and macrophage lineages. For patients with more than one metastasis, selection of the most appropriate metastasis for staining was at the disclosure of the pathologist; typically, the same tissue specimen which was used for diagnostic procedure and therapy response monitoring was included to the study. Clinicopathological characteristics of patients are summarized in Table 1: demographic data, pathologic staging by TNM classification system, number and size of liver metastases, vitality of liver metastases (for panel II), disease free interval, and data on postoperative chemotherapy are provided. Both date of recurrence and either date of death or date last seen were recorded. Overall survival (OS) was defined as the time interval between diagnosis and cancer-related death (with 22 documented cases of cancer-related death); recurrence free survival (RFS) as the time between diagnosis of metastasis and disease progression as estimated by recurrence of metastasis or any type of tumor (with 46 documented cases of disease recurrence). Disease free interval (DFI) was defined as the time between diagnosis of primary colorectal cancer and liver metastases in patients with metachronous development of metastatic disease. Vitality of liver metastases was assessed as described [39].

**Table 1:** Patient characteristics.

	Panel I (n = 14)	Panel II (n = 51)	P*
Median Age (range), years	65.2 (45.8–78.3)	63.8 (38.8–80.4)	0.380
Gender, No. (%)			
Female	6 (42.9)	16 (31.4)	0.527
Male	8 (57.1)	35 (68.6)	
T stage primary tumor, No. (%)			
T0	0 (0)	1 (2.0)	0.521
T1	0 (0)	3 (5.9)	
T2	5 (35.7)	7 (13.7)	
T3	8 (57.1)	30 (58.8)	
T4	1 (7.1)	7 (13.7)	
n.a.	0 (0)	3 (5.9)	
N stage primary tumor, No. (%)			
node negative	5 (35.7)	19 (37.3)	0.794
node positive	9 (64.3)	29 (56.9)	
n.a.	0 (0)	3 (5.9)	
M stage primary tumor, No. (%)			
M0	14 (100)‡	21 (41.2)	n.a.
M1	n.a.	29 (56.9)	
n.a.	0 (0)	1 (2.0)	
Median number of metastases (range)	2 (1–10)	2 (1–10)	0.491
Largest median diameter, cm (range)	2.6 (0.8–7.0)	2.0 (0.3–13.0)	0.345
Median % vital tumor cells (range)	n.a.	25 (0–90)	n.a.
Median disease free interval, months	25.1 (7.9–117.2)	18.0 (0–116.5)	0.166
Type of cytotoxic chemotherapy, No. (%) within 6 months of liver resection			
fluoropyrimidine+oxaliplatin	n.a.	46 (90.2)	n.a.
fluoropyrimidine+irinotecan	n.a.	5 (9.8)	
Regimens containing bevacizumab	n.a.	51 (100)	

*Wilcoxon's rank sum test was used for continuous variables, Chi-square test for ordinal variables.

‡ According to the inclusion criteria for panel I, all patients were M0 at the time of diagnosis of primary tumor.

### Immunostaining on paraffin-embedded tissue sections and staining evaluation

Paraffin-embedded 4 µm-thick sections underwent routine staining with haematoxylin and eosin. Sections of CRCLM specimens were taken for CD45, CD20, AID, IgM, CD138, and CD68. To detect CD45, a common leukocyte antigen, rabbit clonal antibody, clone E19-G (DB Biotech, Kosice, SR) was used. CD20 (mouse monoclonal antibody, clone L26, from Thermo Scientific, Cheshire WA7 1PR, UK or rabbit clonal antibody, clone E17-P, from DB Biotech, Kosice, SR) was used as general B-cell marker. To detect AID, clone ZA001 mouse IgG1-kappa (Invitrogen, Paisley, UK) was used. Anti-AID antibodies were previously shown to be functional in immunostaining of Raji, lymphoblastoid cells derived from a Burkitt lymphoma, and of AID-positive cells in paraffin-embedded tissue sections including tonsils and nasal polyps 24,40,41. To detect IgM, a rabbit polyclonal antibody was used (Acris, Herford, Germany). To detect CD68, monocytes/macrophages lineage marker, mouse monoclonal antibody, clone KP1 (Thermo Scientific, Cheshire WA7 1PR, UK) was used. For immunohistochemistry method, DAKO EnVision+, Peroxidase system (DAKO, Glostrup, Denmark) was applied after the first antibody. Sections were counterstained with haematoxylin for nuclear visualization. A fluorescent staining with anti-mouse and anti-rabbit Ig secondary antibodies conjugated to Alexa dyes 488 and 568 (Invitrogen, Paisley, UK) was used for CD45 staining as well as for double staining (CD20 and CD68). Nuclear counterstaining in this case was performed with DAPI (Roche, Mannheim, Germany). TissueFAXS (TissueGnostics, Vienna, Austria), a fully automated microscopy-based tissue analysis system was used for the acquisition of diseased specimen tissues and quantitative analysis. The TissueFAXS technology allowed to automatically scan the large-scale tissue sections in the fluorescence or bright field modes, enabled to visualize tissue structures followed by identification of the individual cells in the complex native tissue environment and quantitative analysis of positively stained cells [24], [42], [43]. Thus, this approach provided the microscopic images of the entire metastatic tissue sections. Each sample was composed from 100 or more individual fields of view. As representative example, an area of 400 mm^2^ of tissue specimen was typically acquired for follow up analyses. For acquisition the 20x/0.5 objective was used (EC Plan_NeoFluar, Zeiss). Filter sets were from Chroma TechnologyCorp (DAPI 350/460 nm; FITC/Cy2 470/525 nm; TxRed/Cy5 620/700 nm). Quantitative analysis of infiltrating immune cells was performed using TissueQuest or HistoQuest software (TissueGnostics, Vienna, Austria) for immunofluorescent and for immunohistochemical staining, respectively. The evaluation of stained immune cells was conducted without knowledge of the clinical parameters for each patient. To determine the prognostic relevance, ectopic follicles were defined by (i) characteristic compact, round lymphoid aggregate morphology with (ii) a minimal diameter of 20 cells, and (iii) the presence of CD20-positive B cells. Thus, all lymphoid aggregates fulfilling these criteria independent of their activation status were included in the prognostic marker. Ectopic follicular structures were calculated based on comparative analysis of CD45 and CD20 staining patterns for each individual sample by three independent observers with strong expertize in microscopy. All individual staining-derived variables across the specified regions were used for correlation with clinicophathological parameters. For some staining-based variables few samples were excluded from quantification analysis based on tissue quality and/or background staining; for those cases the exact number of specimens is indicated in the figure legends.

### Statistical analysis

In respect of power calculation, under the assumption of a two-sided significance level a hazard ratio of 3.0 can be detected with a power of 90% if 35 events are observed in the sample. Categorical data were described by absolute and relative frequencies. Group differences were tested by chi-squared test or chi-squared trend test for ordinal variables. In case of sparse data exact tests were used. Continuous data were described with median, minimum and maximum. Group differences were assessed by Wilcoxon's rank sum test or by t-test in case of normal distribution (normal distribution was achieved after logarithmic transformation). An analysis of variance was used to compare the three regions (border, portal field, distant liver) within patients. A Tukey adjustment was made for multiple pairwise comparisons. Correlations coefficients were calculated by Spearman's correlation for ordinal variables and Pearson's correlation for log2 transformed continuous variables. OS and RFS were shown by Kaplan-Meier graphs and group differences were tested using log-rank test. For ordinal variables a log-rank test with a trend effect was used. Additionally, hazard ratios and corresponding 95% confidence intervals were estimated by Cox regression models. Staining-derived values were log2 transformed for Cox regression models to achieve approximate normal distribution. Interactions between the two panels and staining-based variables were calculated. In case of non-significant interaction or if both effects for the two panels direct in the same direction, a stratified Cox regression model was used when panel I and II data are analysed in combination. Multivariate Cox regression models were used to adjust the prognostic effects of staining-based variables to clinical parameter(s) found to be significant in univariate Cox regression. Statistical analyses were done using SPSS software version 20 (IBM Corporation, Armonk, New York, USA); all p values given as two-sided and p≤0.05 was considered statistically significant.

## Results

### Algorithm for characterization and quantitative analysis of resident and infiltrating immune cell populations within CRCLM tissue samples

**Figure 1:**
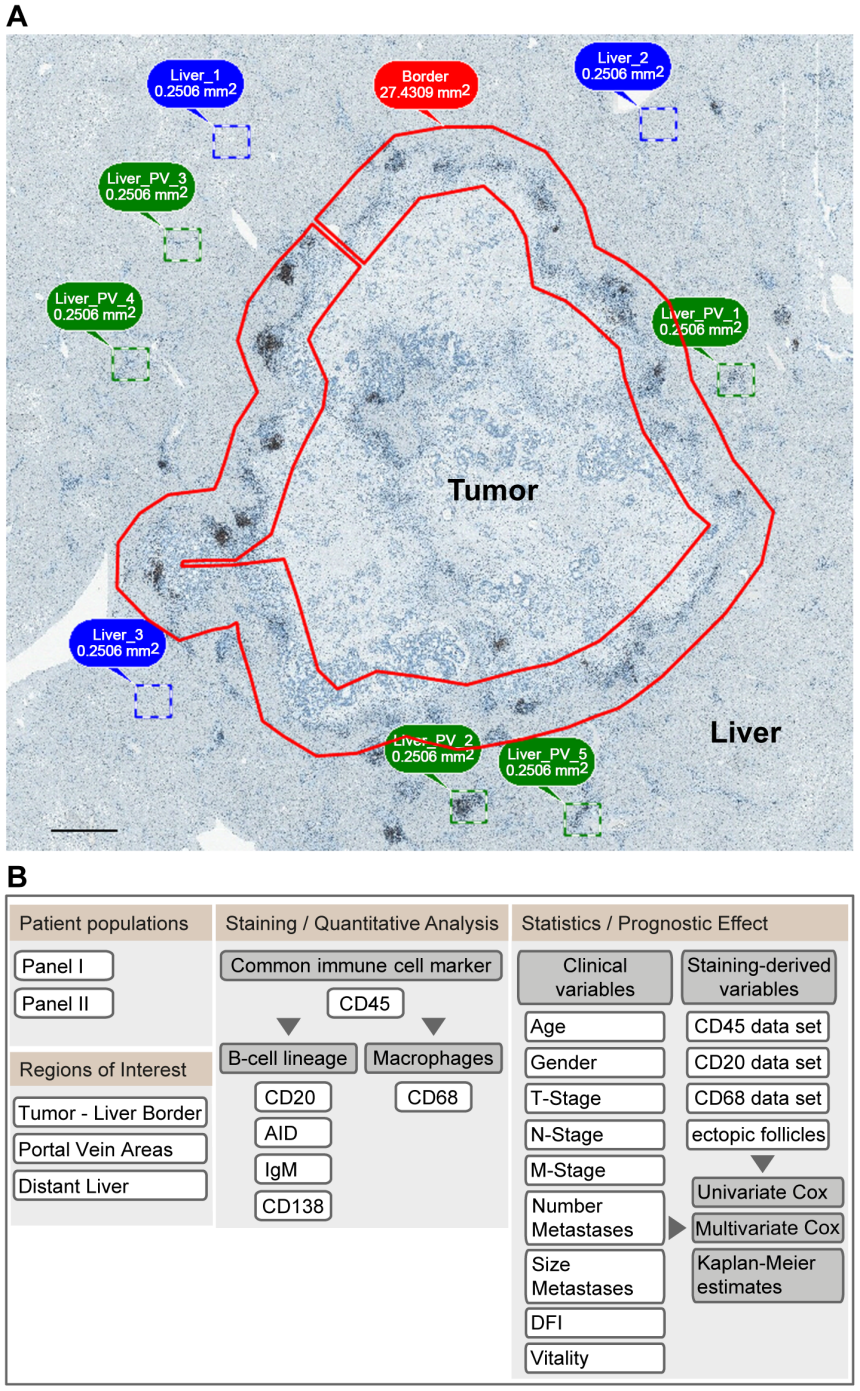
Algorithm for characterization, quantitative analysis, and estimation of the prognostic effect of immune cell populations. (A) Definition of regions of interest within CRCLM tissue samples to unify the quantification strategy between the specimens. Upon acquisition of a large-scale sample using the microscopy-based TissueFAXS system, the regions of interest were defined at the tumor – liver border (indicated by a *red* line), within liver tissue around the portal vein areas (indicated by dashed squares in *green*) and areas within the liver tissues distant to the portal veins (indicated by dashed squares in *blue*). Of note, the herein defined strategy for using 0.5×0.5 mm area for analysis of the tumor – liver border was established empirically based on the microscopic evaluation of CD45-stained sections showing the lymphocyte accumulation within this region; this was specified independent to but matching the quantification area defined by Halama et al [3]. Scale bar: 1 mm. (B) Study design. The study design is summarized by the flowchart including staining with established markers, immune cell organisation pattern, quantification, and assessment of clinical relevance by alignment with clinicopathological parameters.

To analyse and quantify the presence of resident and infiltrating immune cells within the CRCLM tissue we established an algorithm using the TissueFAXS system. For specimen's comparison we applied uniform strategy by making sub-regions at the tumor – liver border (approximately 0.5×0.5 mm on each side of the border with a mean size of about 30 mm^2^) and within the liver tissue around the portal vein areas (five representative areas sized to 0.25 mm^2^) and areas within the liver tissues distant to the portal veins named as distant liver in the follow up descriptions (three representative areas sized to 0.25 mm^2^) (a representative picture is shown in Figure 1, A). Study design is summarized by the flowchart in Figure 1, B. Paraffin-embedded sections were first stained with anti-CD45 antibodies to detect all classes of resident and/or infiltrating immune cells across the patient specimen. Next, to specify the immune cell populations we discriminated between the B cell lineage and the monocyte/macrophage lineage (Figure 1, B). Quantitative analysis using the TissueQuest/HistoQuest software was applied to determine the amount of CD45-positive, CD20-positive, and CD68-positive cells found within the specified above locations of interest. The calculation algorithm is described in Figure S1. To determine the clinical relevance of the patient-specific immune response at site of metastasis, staining-derived data sets were used for alignment with clinicopathological parameters.

### Patient-specific extent and distribution pattern of CD45-positive cells at the site of CRCLM

**Figure 2:**
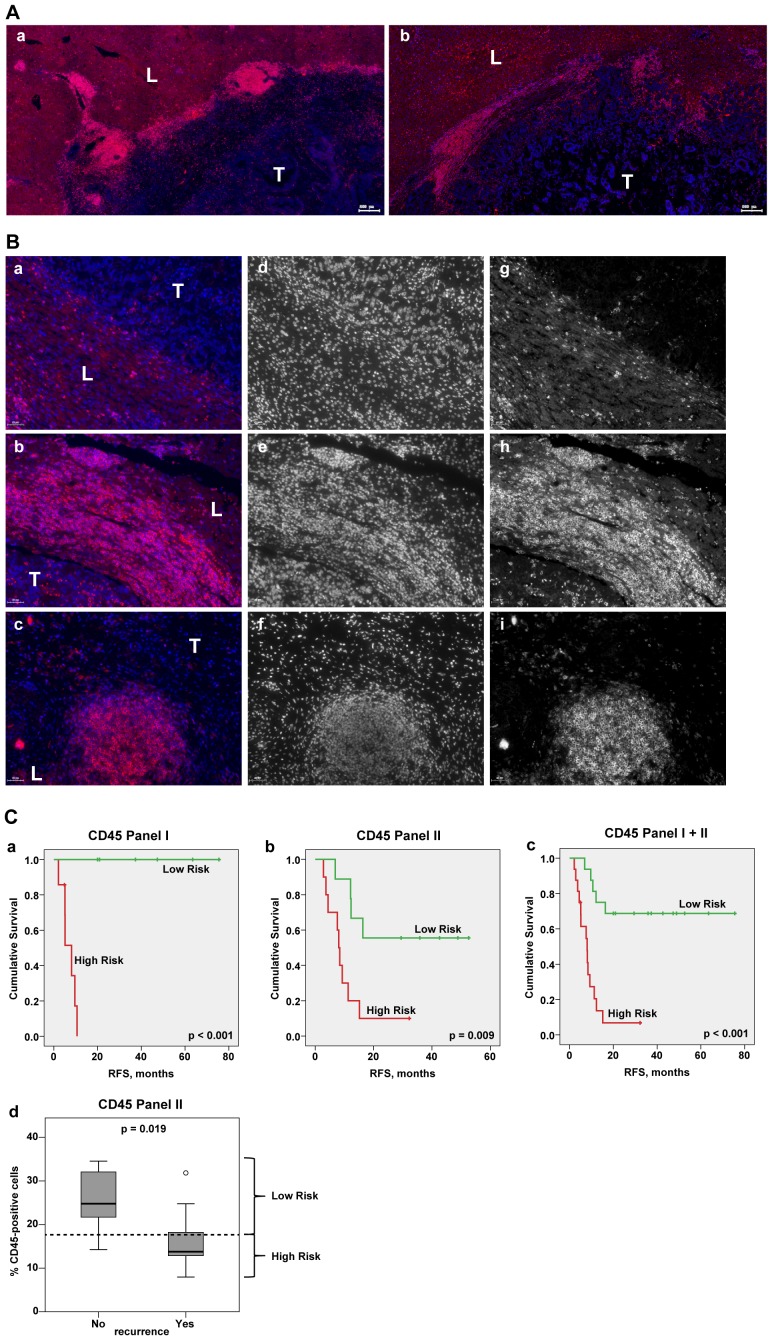
Patient-specific imprint of CD45-positive cells at the site of CRCLM. (A) Massive infiltration of CD45-positive cells confined to the tumor – liver border of the metastases. Representative parts of the metastatic areas for two CRCLM patients are shown (red channel for CD45, blue channel for nuclei/DAPI). T: tumor; L: liver. Scale bar: 200 µm. (B) Different patterns of CD45-positive cell accumulations at the metastatic border. Representative images of various types of immune cell infiltrates are shown: (*a*) single cells; (*b*) large immune cell aggregates; (*c*) prominent ectopic follicle. In addition to the merged images (*a–c*, red channel for CD45 and blue channel for DAPI), pictures of individual channels are included (*d–f* for DAPI; *g–i* for CD45); the individual channels are shown in black/white, whereas merged images are shown in color. T: tumor; L: liver. Scale bar: 50 µm. (C) (*a*–*c*) Kaplan-Meier estimates for patients stratification based on the CD45-derived values at the border. Kaplan-Meier curves for RFS based on CD45 values for panel I, panel II, and their combination are shown giving patients' stratification into low and high risk groups (higher than median indicates low risk); p value of the log-rank test is indicated. Panel I: median is equal to 20.95, below median n = 6, above median n = 7; panel II: median is equal to 17.66, below median n = 10, above median n = 9; panel I+II: median is equal to 19.13, below median n = 16, above median n = 16. (*d*) Boxplots of CD45 data sets for patients without recurrence (No) versus patients with recurrence (Yes) at the time point of 17 months. The cut off was set according to the latest event occurrence (16.3 months) where no censoring has occurred, thereby, providing clear separation from the censored subjects (≥32.2 months) as visualized on (*b*). The median CD45 value, which was used for patient stratification into low and high risk groups on the Kaplan-Meier plot (*b*) is indicated by dashed line; p value is shown (t test).

Gross examination revealed massive infiltration of CD45-positive cells confined to the tumor – liver border of the metastases. Representative images are shown in Figure 2, A. Different patterns of immune cell accumulations were observed among the patients and might coexist within the same specimen at the tumor – liver border: single cells, diffused cell aggregates, and ectopic follicles structures (Figure 2 B). The amount of CD45-positve cells at three regions of interest exhibited major inter-patient variability as determined by quantitative analysis: at the border ranging from 8% to 46% (median 19%), within the liver tissue at site of portal veins ranging from 9% to 56% (median 29%) and within the distant liver tissue ranging from 4% to 16% (median 9%); the analysis was performed for panel I and panel II together. Importantly, CD45-derived data sets showed no significant differences between panel I and panel II (Figure S2).

### CD45-positive resident and/or infiltrating lymphocytes within CRCLM tissues: prognostic effect

To determine the clinical relevance of the patient-specific immunological imprint, the CD45-derived data sets were correlated with recurrence free survival (RFS) and overall survival (OS). As estimated by Cox regression analysis (Table S1), the presence of CD45-positive cells at the tumor – liver border was found to show a strong positive prognostic effect on RFS in both patient panels (panel I: HR = 0.004, 95% CI: <0.001–0.627, p = 0.033; panel II: HR = 0.156, 95% CI: 0.045–0.546, p = 0.004) and for panel I and panel II together (HR = 0.093, 95% CI: 0.028–0.305, p<0.001); the combination of data sets was possible based on additional testing for interactions between the two panels and staining-based variables (Table S2). Furthermore, a higher CD45 content at the metastatic border was found to correlate with better OS, although with weaker prognostic effect (Table S1). With respect to RFS, patients were sub-divided into groups with a higher percentage of CD45-positive cells (above the median) and with a lower percentage of CD45-positve cells (below or equal to the median). Figure 2, C shows the corresponding Kaplan-Meier graphs. Separate analyses of panel I and panel II as well as their combination showed statistically significant differences between high and low risk groups (log-rank test: p<0.001, p = 0.009, and p<0.001, respectively). As no events are present in the low risk group of panel I (n = 7), the corresponding HR of Cox regression analysis tends to infinity. Additionally, for panel II data, distribution of CD45-based values can be shown for patients with and without disease recurrence at 17 months, when no censoring has occurred yet (Figure 2, C, *d*). Patients without disease recurrence showed significantly higher CD45 content in comparison to those with a recurrence (p = 0.019). In contrast to the border, alignment of liver-derived data sets (portal vein areas and distant liver) did not reveal statistically prognostic effect on RFS or OS (Table S1).

In summary, our findings emphasize a strong impact of the patient-specific local immune response taking place at site of metastasis on the clinical outcome of CRCLM patients. It gives us the motivation to delineate the impact of the individual immune cells subsets comprising the CD45-positive cell population using a larger cohort of patients. Accordingly, in the follow up experiments specifying the B-cell and macrophage lineages an enlarged cohort of patients within panel II was investigated (n = 51).

### Patient-specific extent and organisation pattern of CD20-positive B lymphocytes at the site of CRCLM

**Figure 3:**
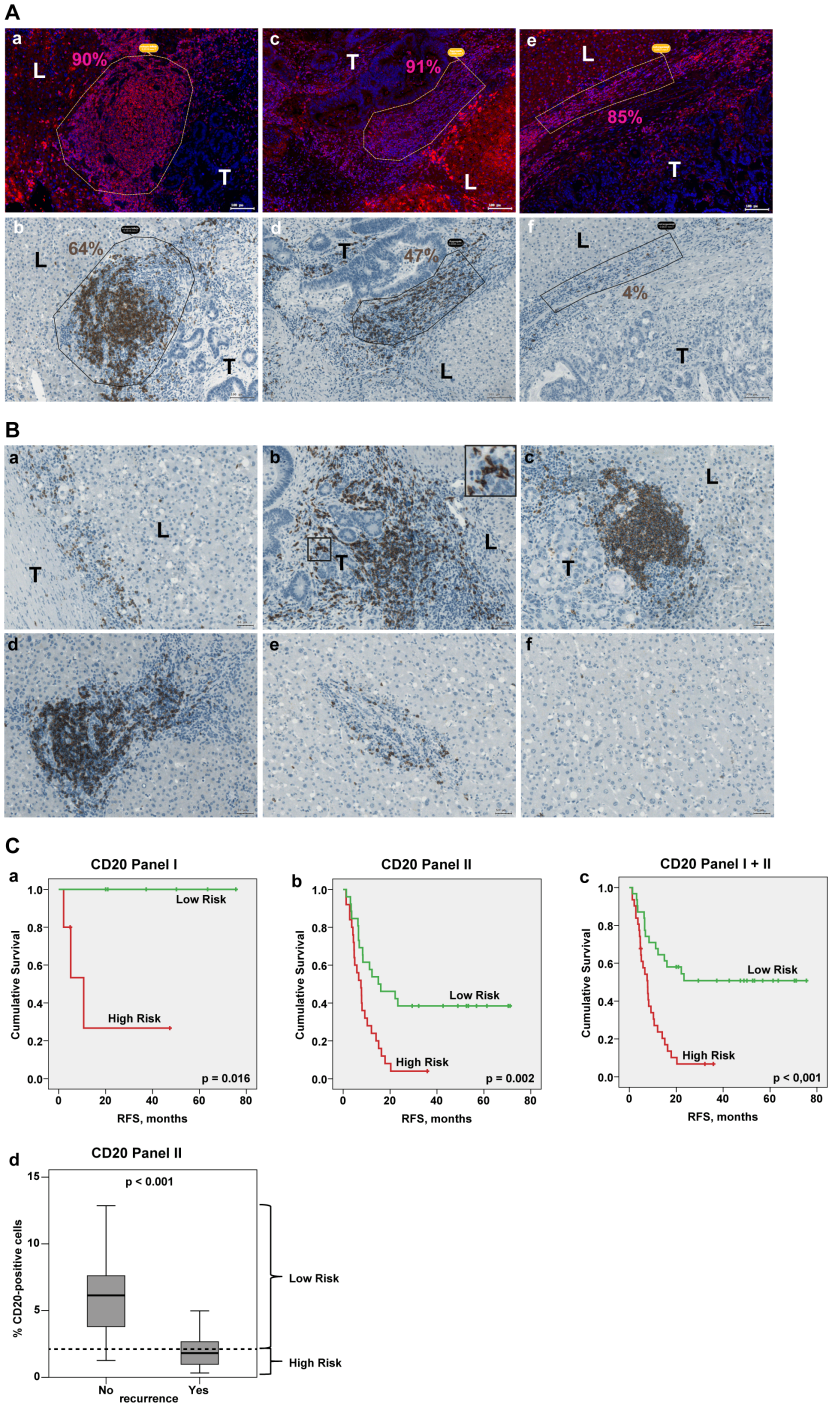
CD20-positive B lymphocytes at the site of CRCLM. (A) Subpopulation of CD20-positive B cells at metastatic border. Representative examples of the CD45-positive (*a*) ectopic follicle, (*c*) large cell aggregate and (*e*) non-organized cell population and the corresponding areas stained for CD20 are shown (*b*, *d*, *f*, respectively). Quantitative analysis was done using TissueQuest and HistoQuest software; percentage of positive cells is indicated. Scale bar: 100 µm. (B) Different organisation patterns of CD20-positive cells within the three regions of interest. Representative images (i) at the border: (*a*) single cells, (*b*) cell aggregates; insert: higher power view of CD20-positive cells in contact with the colon tumor epithelial cells, (*c*) ectopic follicular structures; (ii) around the portal veins (*d*) proximate to the border and (*e*) distant to the border as well as (iii) within liver tissue (*f*) are shown. T: tumor; L: liver. Scale bar: 50 µm. (C) Kaplan-Meier estimates for patients stratification based on the CD20-derived values at the border. Kaplan-Meier curves for RFS based on CD20 values for panel I, panel II and panel I+II are shown giving patient stratification into low and high risk groups (higher than median indicates low risk); p value of the log-rank test is indicated. Panel I: median is equal to 2.70, below median n = 5, above median n = 6; panel II: median is equal to 2.11, below median n = 25, above median n = 26; panel I+II: median is equal to 2.22, below median n = 31, above median n = 31. (*d*) Boxplots of CD20 data sets for patients without recurrence (No) versus patients with recurrence (Yes) at the time point of 24 months. The cut off was set according to the latest event occurrence (23.3 month) where no censoring has occurred, giving separation from the censored subjects (≥29.4 months) as visualized on (*b*). Dashed line: the median CD20 value, which was used for patient stratification into low and high risk groups on Kaplan-Meier plot (*b*); p value is shown (t test).

Next, to characterize the B-cell lineage at CRCLM site, the tissue specimens were analysed for CD20-positive cells. Strikingly, the major fraction of cells within the large CD45-positive cellular aggregates and particularly within the ectopic follicles was found to be CD20-positive B cells; interestingly, only trace amounts of CD20-positive B cells were detected within the non-organized CD45-positive lymphocyte populations (Figure 3, A). Different organisation patterns of CD20-positive cells were observed within the three regions of interest. Representative images are provided in Figure 3, B. At the border, single cells (Figure 3, B, *a*) and/or cell aggregates (Figure 3, B, *b*), and/or ectopic follicles (Figure 3, B, *c*) were detected. Of note, CD20-positive B cells were found to accumulate around the tumor islands (Figure 3, B, *b*, insert). Large B-cell aggregates were as well detected around the portal veins in close proximity to the border area, whereas at more distant portal veins single CD20-positive cells were observed (Figure 3, B, *d*, *e*, respectively). Notably, within distant liver tissue, across the whole specimen only sparsely distributed, single events were found (Figure 3, B, *f*). In line, quantitative evaluations of three regions revealed the presence of B cells at the tumor – liver border ranging from 0.3% to 13% with a median value of 2% and within the liver tissue around the portal veins ranging from 0.1% to 36% with a median value of 4%; single CD20-positive cells were detected within distant liver tissue (median of 0.1%); analysis was performed for panel I and II in combination. Additionally, comparing the two chemotherapy groups within panel II we observed no major difference in the distribution of the staining-derived values and have no indication for group differences between chemotherapies (Table S3). Importantly, comparison of CD20 variables for panel I and panel II revealed no significant differences for three regions of interest (Figure S3).

### CD20-positive lymphocytes at the tumor – liver border of CRCLM: prognostic effect

Univariate Cox regression analysis (Table S4) revealed significant correlation between the percentage of CD20-positive cells at the tumor – liver border and RFS for panel I (HR = 0.125, 95% CI: 0.036–0.432, p = 0.001), for panel II (HR = 0.567, 95% CI: 0.434–0.740, p<0.001) and their combination (HR = 0.534, 95% CI: 0.411–0.693, p<0.001). Furthermore, the amount of CD20-positive cells at the border was found to be significantly associated with OS for the combination of panel I and II with a slightly smaller effect than for RFS (HR = 0.640, 95% CI: 0.431–0.950, p = 0.027). Next, the patients were sub-divided into two groups: patients with a high percentage of CD20-positive cells at the tumor – liver border (above the median) and with a low percentage of CD20-positive cells (below and equal to the median). RFS probabilities for both groups are shown by Kaplan-Meier graphs and differences are tested by log-rank test (Figure 3, C). In both panels and their combination, patients with higher CD20 values have significantly lower risk for RFS than patients with lower CD20 values (panel I, log-rank test: p = 0.016; panel II, log-rank test: p = 0.002; panel I+II, log-rank test: p<0.001). Similarly to the strategy described for CD45-derived data sets, the patient cohort from panel II can be additionally separated by presence or absence of disease recurrence at 24 months, when no censoring has occurred yet. Significantly higher CD20 content was observed in patients without disease recurrence at 24 months compared to those with recurrence before 24 months (p<0.001; Figure 3, C, *d*). Overall, our data demonstrate a positive correlation between the magnitude of B-cell infiltration at the tumor – liver border and clinical outcome for CRCLM patients.

### Ectopic follicular structures assemble within the metastatic margin of CRCLM

**Figure 4:**
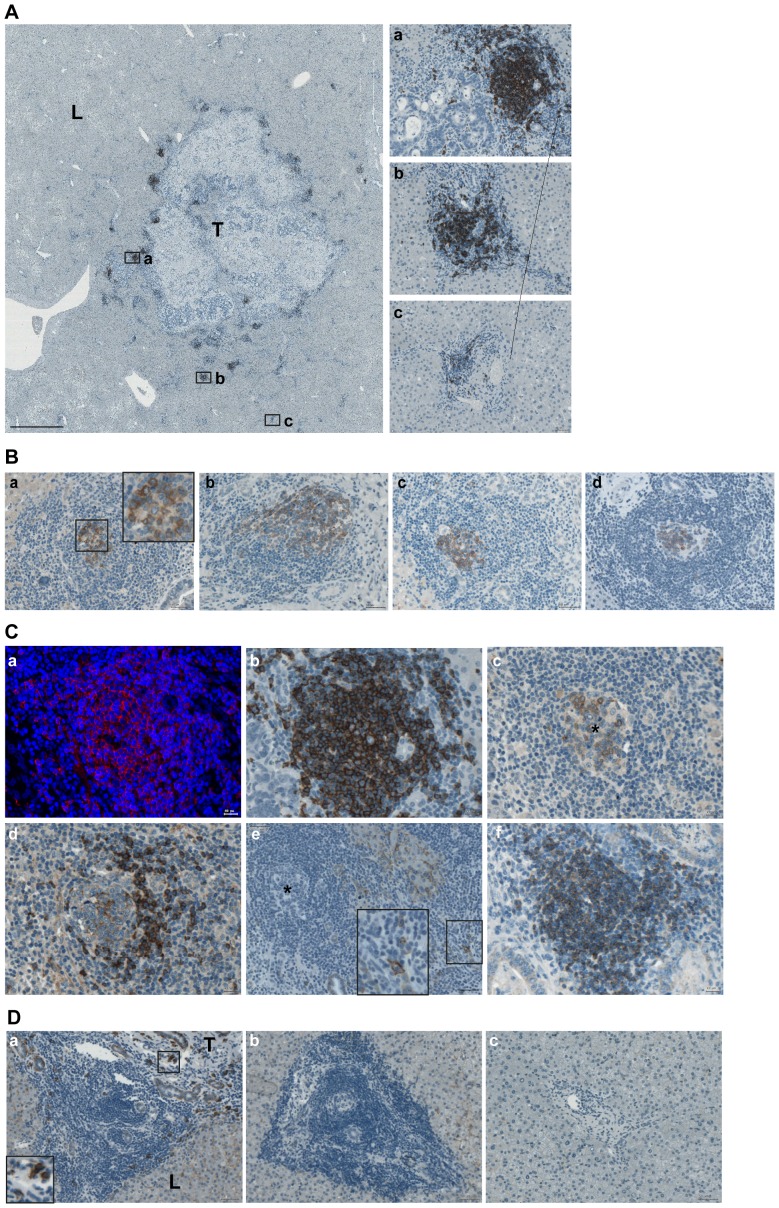
Ectopic follicular structures at the site of CRCLM. (A) When formed, ectopic follicles are attracted to the tumor – liver interface. Representative image of the large-scale metastatic area surrounded by ectopic follicles is shown; brown color, CD20 staining; blue color, nuclear counterstaining with haematoxylin. Scale bar: 2 mm. The higher-power views of follicles at border (*a*), proximal (*b*) and distant (*c*) to tumor portal veins are given. Scale bar: 50 µm. (B) Representative images of AID-positive ectopic follicular structures located at the tumor – liver border are shown (*a*, insert: the higher-power view); brown color, AID staining; blue color, nuclear counterstaining with haematoxylin. Scale bar 50 µm. (C) The characteristic structure of a mature, AID-positive lymphoid follicle (*a-e*) is shown. Consequent slides were stained for CD20 using immunofluorescent (*a*, merged, red color, CD20 staining; blue color, DAPI; CD20 gradient is visible with reduced intensity at the periphery of follicle) and immunohistochemical (*b*, brown color, CD20 staining; blue color, nuclear counterstaining with haematoxylin) procedure; AID (*c*), IgM (*d*), CD138 (*e*). Insert: the high-power view of CD138-positive cells around follicular structure. To visualize distribution of CD138-positive cells, the image (*e*) was reduced by 60% in comparison to those shown on *a-d*. The corresponding AID-positive core of the follicle is indicated by asterisk (*c*, *e*). Example of follicle which is predominantly composed of IgM-positive B-cell subset (*f*, brown color, IgM staining; blue color, nuclear counterstaining with haematoxylin). Scale bar: (*a-d, f*) 20 µm, (*e*) 50 µm. (D) CD138-positive plasma cells were not detected at portal vein areas distant to the border. Additional example of (*a*) follicular structure at border surrounded by CD138-positive plasma cells, (*b*) aggregate of infiltrating cells or (*c*) diffused cells around the portal veins distant to border which do not contain CD138-positive cells; brown color, CD138 staining; blue color, nuclear counterstaining with haematoxylin. Scale bar: 50 µm.

Important observation of the current study describes the presence of ectopic lymphoid follicles at site of the tumor – liver border. Such structures ranged from small aggregates (appr. 20 cells in diameter) to highly organized structures containing blood vessels, ductules, and bile ducts as determined by visual analysis of tissue morphology assessed on HE-stained images and staining of cytokeratin 19 (CK19) known to be expressed by bile duct epithelial cells (Figure S4). Ectopic follicles were detected in 12 out 14 patients of panel I and in 33 out of 51 patients of panel II. The presence of follicular structures was documented using a scoring system (0 – no follicular structures; 1 – low number of follicular structures with n ≤ 2, and 2 – high number of follicular structures with n>2). Comparison of panel I and panel II regarding the ectopic follicle score at the tumor – liver border revealed no statistically significant differences (Figure S5). Furthermore, similarly to the CD20-based results, no major differences were detected in the distribution of the ectopic follicle scores between the two chemotherapy groups of panel II (Table S3). Ectopic follicles could only be detected directly at the tumor – liver border or at the portal veins located in the close proximity, whereas typically no follicles were present within distant liver tissue (Figure 4, A).

To further characterize ectopic follicular structures, consecutive tissue sections were stained with anti-AID to define fully active follicular structures with ongoing CSR and/or SHM events, anti-IgM to detect the naïve B-cell subpopulation and document the presence of the mantel zone in fully established GC-like follicular structures, and anti-CD138 to monitor the differentiation to antibody-secreting plasma cells. Importantly, some ectopic lymphoid structures were found to be AID-positive (Figure 4, B); the presence of AID-positive follicles could be detected in specimens of patients with and without chemotherapy. Characteristic fully developed ectopic follicles were composed of the CD20-positive body including the AID-positive core structure surrounded by the IgM-positive mantel zone (Figure 4, C, *a*–*d*). CD138-positive plasma cells were detected in a close proximity to AID-positive follicles (Figure 4, C, *e*). In addition, follicles predominantly composed of naïve B-cell subset as determined by anti-IgM staining were observed (Figure 4, C, *f*); however, such type of follicles comprised only a minor population and might represent the early stages of ectopic follicular structure development being on their way to mature follicles. No CD138-positive plasma cells could be detected at distant proximity to metastasis (Figure 4 D).

### Ectopic follicular structures within the metastatic margin of CRCLM: prognostic effect

**Figure 5:**
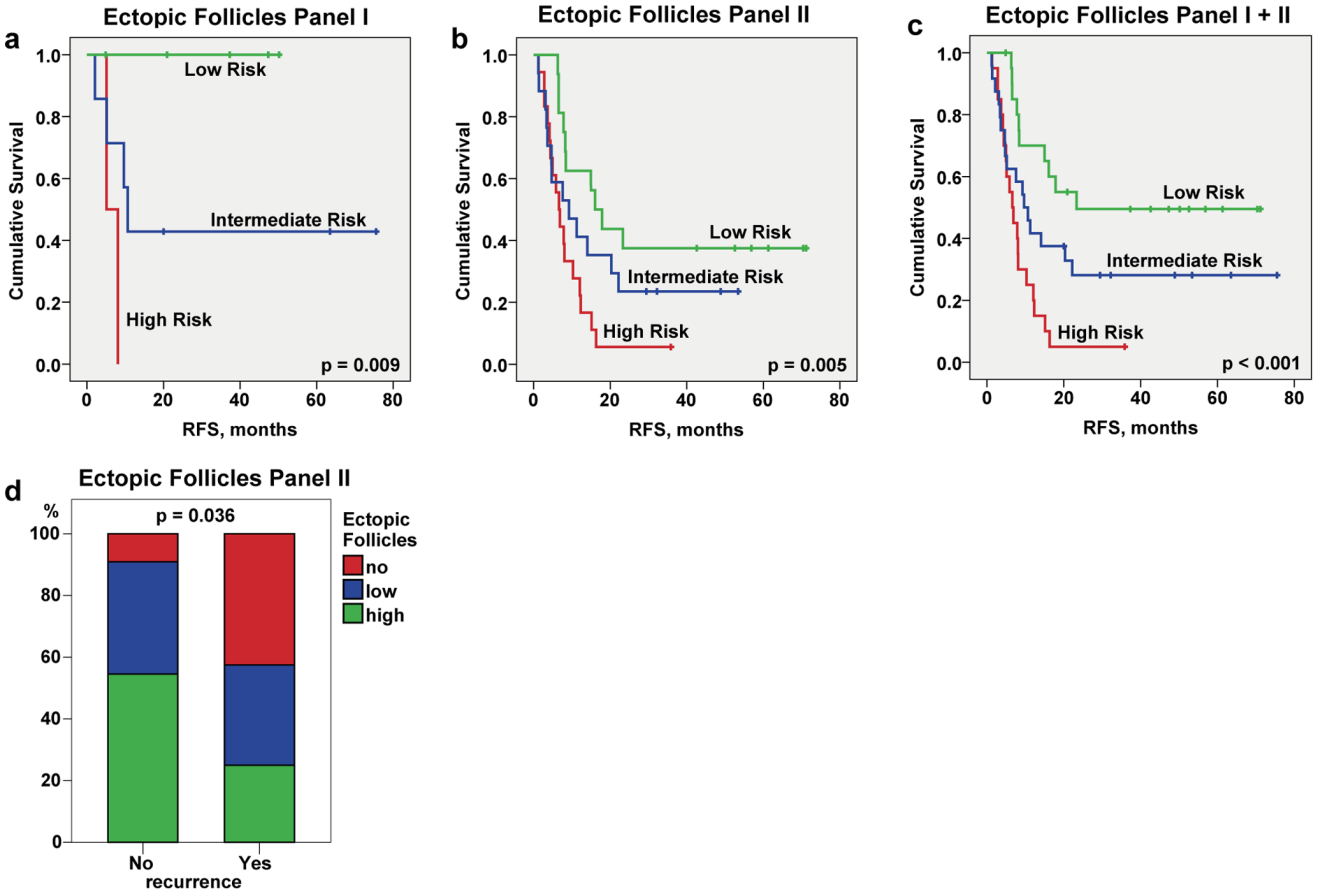
Prognostic effect of ectopic follicular structures allocated at the tumor – liver border. Kaplan-Meier estimates for patients stratification based on the ectopic follicle score at the border (*a*–*c*). Kaplan-Meier curves for RFS for panel I, panel II, and panel I+II show patient stratification into low (high number of follicular structures), intermediate (low number of follicular structures) and high risk (no follicular structures) groups; p value of the log-rank trend test is indicated. Panel I: low risk n = 5, intermediate risk n = 7, high risk n = 2; panel II: low risk n = 16, intermediate risk n = 17, high risk n = 18; panel I+II: low risk n = 21, intermediate risk n = 24, high risk n = 20. (*d*) Comparative assessment of contribution of *no*, *low*, and *high* ectopic follicle score to the total quantity for patient sub-groups without (No) and with (Yes) disease recurrence as estimated at the time point of 24 months where no censoring has occurred; p value of chi-square trend test is shown.

Next, we raised the question whether the magnitude of follicles developed within the border, designated as ectopic follicle score, is associated with clinical outcome. A higher score for ectopic follicular structures at the tumor – liver border showed significant better prognostic effect on RFS for patient samples from panel I and panel II when analysed individually and in combination (Table S5; univariate cox regression; panel I: HR = 0.145, 95% CI: 0.029–0.729, p = 0.019; panel II: HR = 0.575, 95% CI: 0.387–0.856, p = 0.006; panel I+II: HR = 0.520, 95% CI: 0.354–0.765, p<0.001). Figure 5 illustrates the results of the log-rank test for RFS showing significant patient stratification into low, intermediate, and high-risk groups (panel I, log-rank test: p = 0.009; panel II, log-rank test: p = 0.005; panel I+II, log-rank test: p<0.001). Additionally, a significant shift was seen towards a higher follicle score for patients of panel II without disease recurrence (no event before 24 months) in comparison to those with a disease recurrence before 24 months (p = 0.036; Figure 5, *d*).

### Evaluation of CD68-positive macrophages at site of metastasis

**Figure 6:**
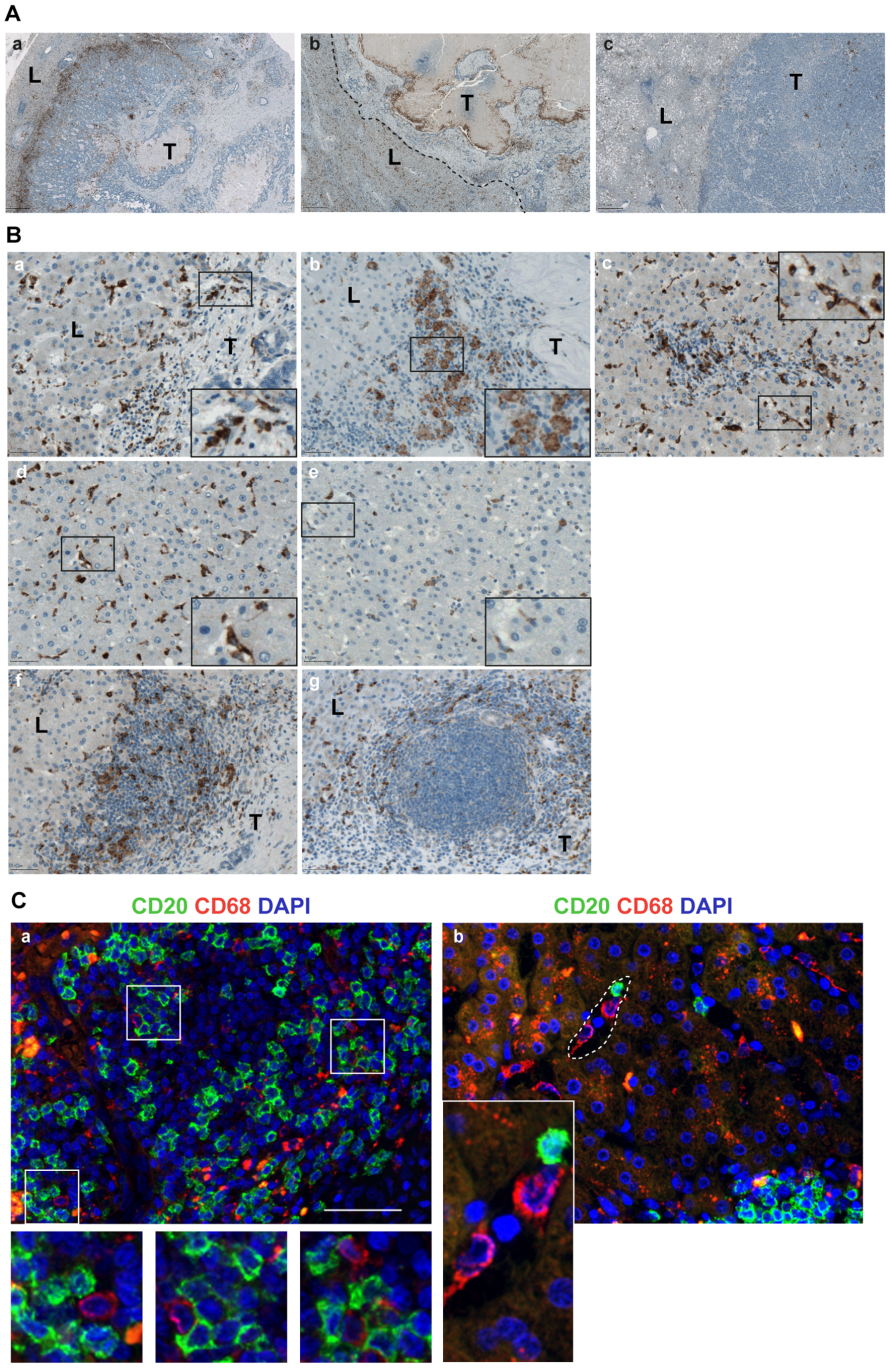
Localization patterns of CD68-positive macrophage populations across CRCLM specimens. (A) Representative images of three major distribution patterns are shown: (*a*) accumulation of CD68-positive macrophages lining the tumor body within the tumor – liver border sub-region; (*b*) CD68-positive rim bounding tumor parts and differentiating them from necrotic areas within the metastatic body; tumor – liver margin is indicated by dashed line; (*c*) no preferential accumulation either at the border or tumor. Brown color, CD68 staining; blue color, nuclear counterstaining with haematoxylin. Scale bar 500 µm. T: tumor; L: liver. (B) Various subpopulations of CD68-positive lymphocytes (*a, b*) at the tumor – liver border and (*c*) within the portal vein area characterized by well-spread or round shaped morphology are shown; in addition, (*d*, *e*) resident CD68-positive Kupffer cells within distant liver shows inter-patient variability regarding intensity of staining and density; representative images of CD68-positive cells within (*f*) large immune cell aggregates and (*g*) ectopic follicles. Scale bar 50 µm. Inserts: the high-power views. T: tumor; L: liver. (C) Double immunofluorescent staining to visualize the co-localization of CD20-positive B cells and CD68-positive macrophages within the tumor – liver sub-region. The merged images are shown (green channel for CD20, red channel for CD68, and blue channel for DAPI): (*a*) direct cell-cell contact between CD20- and CD68-positive lymphocytes; (*b*) CD20-positive B cells might co-localize with the resident CD68-positive Kupffer cells within vascular sinusoids; example of such sinusoid is highlighted by a dashed line. Scale bar 50 µm. Inserts: the high-power views.

Microscopic evaluation of whole tissue specimens revealed strong inter-patients variability regarding spatial distribution of CD68-positive cells. Three major distribution patterns could be observed as summarized in Figure 6, A: CD68-positive macrophages strongly attracted to the tumor – liver interface forming a clear rim at the metastatic border (32 out of 62 patients); no accumulation of CD68-positive cells at the tumor – liver border, however, apparent CD68-positive layer bounding the tumor islands within the partially necrotic metastatic body (15 out of 62 patients); low number and homogenous distribution of CD68-positive cells within border, liver, and tumor without any preferred place of accumulation throughout the tissue sample (15 out of 62 patients). Of note, in some patients mixed distribution patterns were observed; thus, the numbers should be considered as approximate estimations. Furthermore, detailed analysis of three regions of interest revealed heterogeneity in morphology and staining intensities among the patients. CD68-positive macrophages characterized by their elongated, well-spread morphology as well as round shaped phagocytes were detected at the tumor – liver border both from the side of the tumor body and the liver (Figure 6, B, *a*, *b*) and within the portal vein areas (Figure 6, B, *c*). Homogenously distributed CD68-positive resident Kupffer cells detectable within sinusoids in the liver showed inter-patients variability regarding morphology and staining intensity (Figure 6, B, *d*, *e*) likely reflecting their activation status. Additionally, large cellular aggregates and ectopic follicular structures were found to be intercalated and/or surrounded by CD68-positive cells (Figure 6, B, *f*, *g*). In line, as demonstrated by double staining, CD68-positive macrophages were observed in close proximity to and/or directly attached to CD20-positive B cells at the border (Figure 6, C). In line with visual observations, quantitative evaluations of three regions of interest revealed the CD68-positive cells at the tumor – liver border ranging from 2% to 24% (median 5%), within the liver tissue around the portal veins ranging from 1% to 18% (median 3%), and within distant liver tissue ranging from 1% to 10% (median 3%); analysis was performed for panel I and II in combination. Results revealed no significant differences for panel I and panel II within three regions of interest (Figure S6). Additionally, there are no indications for group differences between chemotherapies within panel II (Table S3). CD68-positive macrophages were significantly increased at portal fields and even stronger at the border compared to the distant liver (p<0.001 and p<0.001, respectively). However, despite strong inter-patient variation in the CD68-attributed immunological imprint, the univariate Cox regression analysis did not show significant prognostic effects for both RFS and OS (Table S6).

### Correlations between staining-derived variables from three regions of interest

Correlation analysis was performed for staining-derived variables in the three regions of interest. As summarized in Table S7, highly correlated parameters (r>0.5; all with p≤0.002) were found (i) between CD45_Liver Portal Vein and both, CD45_Border and CD45_Liver Distant; (ii) between CD20_Border and CD20_Liver Portal Vein; (iii) between all three CD68 regions (CD68_Border, CD68_Liver Portal Vein and CD68_Liver Distant). Furthermore, highly positive correlations could be observed between CD20_Border and both, CD45_Border and ectopic follicles.

### Prognostic effects of CD45-, CD20-, and ectopic follicles-based variables in context to clinical parameters

Effects of clinical parameters such as age, gender, TNM stage, number and size of metastases, DFI, and vitality of metastases on RFS and OS were assessed using univariate Cox regression (Table S8). Notably, only vitality showed weak significant effects on RFS (HR = 1.013, 95% CI: 1.001–1.025, p = 0.029) and on OS (HR = 1.020, 95% CI: 1.003–1.037, p = 0.023). Next, using multivariate Cox regression analyses for panel II data, prognostic effects of the staining-based variables were adjusted for vitality. CD45 content at the border, CD20 content at the border, and the ectopic follicle score still remained as independent prognostic factors for RFS (Table S1, S4, and S5, respectively: columns *multivariate adjusted for vitality*).

## Discussion

Understanding the relationship between tumor characteristics and immune response evolved locally or systemically is now proposed to be considered as integral part of the clinical management of several cancer types (reviewed in [4], [6]–[8] and recent [44], [45]). This approach is highly and equally relevant for cancer immunotherapy as well as patient stratification for prognostic and predictive monitoring. In the current study we suggest the patient-specific immunological imprint of B lymphocytes locally at the tumor – liver border to be used as novel prognostic marker in CRCLM patients.

The characteristic feature of CRCLM tissue sample with clearly visible metastatic area and metastatic border upon tissue staining allowed us to standardize an algorithm for quantification within the specimen and across the specimens to cover both the intra-patient heterogeneity and the inter-patient variability. Herein established strategy provides superior solution for unbiased large-scale sample' quantitative analysis which is, by our opinion, prerequisite for both biologically and statistically relevant evaluations when patient-specific immunological imprint has to be assessed.

The CD45-based results are strongly suggestive for active participation of attracted leukocytes in the mechanisms preventing tumor progression. Potential limiting factor for CD45 to be used as marker in clinics, we assume, might be attributed to the differences in staining intensities and cellular morphologies of various immune cell lineages comprising the entire CD45-positive leukocyte population. Of particular importance, besides patient-specific characteristics regarding extent of CD45-positive leukocytes, the CD45-based staining was strongly suggestive for the phenomenon of ectopic lymphoid follicle formation taking place at the tumor – liver interface. This was an indirect indication to the presence of B cells at metastatic border as main constituent of GC-like ectopic follicles.

Indeed, herein we report for the first time the presence of CD20-positive B lymphocytes which are confined to the tumor – liver border of CRC metastasis. The CD20-positive B cells, as expected, were detected as dominant population within CD45-positive follicular structures and additionally within large cellular aggregates at the border and those portal fields which are allocated in close proximity. Surprisingly, no or only single CD20-positive cells were detected within distant liver tissue potentially excluding B-cell recruitment from the blood through the hepatic sinusoids.

We show a strong prognostic value of the CD20-based data sets. Overall, our data propose the extent of CD20-positive cells at the tumor – liver border as novel independent prognostic marker for survival which is superior to any clinical parameter. This fact furthermore emphasizes the biological relevance of the herein applied quantification strategy for CD20-positive lymphocytes by defining the 0.5×0.5 tumor – liver border region.

What is currently known about the CD20 molecule? CD20 is a classical marker of B-cell identity, which is expressed on pre-B lymphocyte and mature B cells and down-modulated upon differentiation to plasma cells [46]. Interestingly, being the first identified B-cell-specific marker, CD20 functionality has remained unclear and thus far no natural ligand for CD20 has been identified. Thus, despite the success in immunotherapy using anti-CD20 monoclonal antibodies for lymphoma patients, knowledge about the biology of CD20 is still relatively limited. Accumulating evidence indicates that CD20 expression can be controlled at transcriptional, posttranscriptional, and posttranslational levels [47], [48]. Furthermore, few examples point out that CD20 expression can be modulated by known therapeutic agents [49]–[51].

An additional important finding of the current study is the identification and characterization of ectopic follicular structures confined to the border of CRC metastasis. Evaluation of ectopic follicular structures revealed strong inter-patient variability dependent on the presence and, if detected, on the number and size of follicles as well as their activation status/functionality estimated by AID expression and detection of CD138-positive plasma cells. In general, ectopic follicular structures are known to have many but not necessarily all of the GC characteristics, since the mechanisms driving B cells to form follicular structures with specialized functions are diverse and seem to be dependent on the microenvironment within the diseased tissue [23], [24], [30], [52].

Why are ectopic follicles formed at the boundary between the metastasis and hosting liver tissue? Although the mechanism(s) are not yet defined, the findings of the study support the notion that the microenviroment at the colon tumor – liver interface creates the key conditions for B-cell assembly into ectopic follicles and provides the functional niche allowing B cells to undergo affinity maturation and clonal selection. Additionally, some of the expanded B-cell clones might get a memory phenotype and enter the circulation and/or, as shown herein, differentiate to (long-lived) CD138-positive plasma cells maintaining certain levels of pre-selected high-affinity antibodies. Interestingly, highly organized ectopic follicular structures are characterized by the presence of embedded bile ducts. Although very limited information is available, the accumulation of inflammatory immune cells, predominantly macrophages and plasma blasts, around the bile ducts was recently documented in an early stage of patients with primary biliary cirrhosis [53]. In line with our results, this might suggest that cytokines, chemokines or other active molecules secreted by the biliary epithelial cells mediate chemoattraction and/or activation of immune cells. Intriguingly, bile acid was shown to be able to trigger AID expression via NFκB activation in oesophageal squamous-derived cells during the development of Barrett's oesophageal adenocarcinoma [54]. Hence, although speculative, the role of hepatic and/or intestinal bile acid(s) as an active mediator contributing to B-cell migration and/or maturation and/or follicle assembly at the CRCLM site cannot be excluded.

In line with CD20-attributed results, the data strongly suggest that the patient population whose metastatic tumors are surrounded by ectopic follicular structures have a lower risk of recurrence and thus propose that the ectopic follicles at the metastatic border might be considered as additional prognostic marker to estimate the recurrence of disease shown to be superior to clinical variables. In contrast to the herein described computerized calculations of the CD20-positive B cells, we used a scoring system for assessment of the prognostic relevance of ectopic follicles. Although the scoring-based assessment had a strong prognostic power and a certain benefit for easy translation to the clinics, a standardization of “ectopic follicles” as variable allowing an observer-independent, computerized quantification might be considered. Among potential marker candidates is the CD21L molecule, specifically expressed by follicular dendritic cells within GCs [23], [55] or DC-Lamp (CD208) of mature DCs detected within ectopic follicles in primary lung tumor tissues and shown to have prognostic effect [29], [56].

Our work is complementary to recent studies [3] highlighting the contribution of cytotoxic T-cell populations to anti-tumor mechanisms which are triggered locally in the liver around the CRC metastasis and proposing their prognostic relevance. Overall, the data emphasize the anti-tumoral role of the adaptive immune response established locally at the tumor – liver interface and do not exclude a synergistic contribution of systemic memory B cells. The important finding that ectopic follicles were detected in both patient groups (without and with chemotherapy) suggests the tumor-associated B-cell-driven immune response to be established before treatment and persist after applying the chemotherapy. Furthermore, given that GC-like ectopic follicular structures are known to be formed in response to and as consequence of antigen stimulation, it is then suggestive that required liver- and/or tumor-associated antigen(s) were present in examined subgroup of patients even before chemotherapy application.

The data trigger few critical questions which need further experimentation to be resolved: (i) is there a dominant clone of B cells either specific for each individual patient and/or common in all those patients with established GC-like structures at the border; (ii) whether only those patients having right antigen are able to build up fully developed ectopic follicles and thus produce the tumor-suppressive antibodies locally and/or systemically; and, finally, (iii) what is the driving antigen. Assessment of clonality of B lymphocytes represents an initial step towards understanding the nature of the antigen. Based on the proposed anti-tumoral role of the CD68-positive macrophage population in patients with primary CRC [57] and considering that liver provides the natural environment for proper functionality of the resident CD68-positive Kupffer cells, we extended our study by creating the CD68-attributed immunological imprint at CRCLM site and assessing the feasibility for disease-triggered cross-communication between B lymphocytes and macrophage lineage. We show that the tumor-driven infiltration process of the monocyte/macrophage population directed to the metastatic area indeed take place. However, clearly observable accumulation at the tumor – liver border as seen for CD20-positive B cells was detected only in subgroup of patients. The B-cell – macrophage direct contacts/co-localizations were shown within large cellular aggregates and ectopic follicles, as well as in the vascular sinusoids. The data suggest that bi-directional instruction for B lymphocytes and macrophages might take place at the tumor – liver interface. Surprisingly, despite strong inter-patient variability in both distribution pattern and magnitude of CD68-positive cells, which were suggestive for patient-specific characteristics regarding clinical outcome, the CD68-derived data sets did not reveal a prognostic relevance. Of note, special characteristics of the macrophage lineage attributed to their heterogeneity in cellular morphology are not covered by thus far available and the herein applied quantification algorithm, thus, representing a potential limiting factor for estimation of prognostic relevance of CD68-based data sets. Aside from that, by using CD68 as a marker we detected the entire macrophage population without discriminating the classical M1 and alternative M2 phenotypes.

Given that the CD68-positive leukocyte subset which is a part of the overall CD45-positive immune cell population likely contributes as diluting factor to the prognostic power of CD45-based imprint, we suggest CD20 and ectopic follicles as prognostic markers. We propose to validate those markers further on independent, larger cohort of CRCLM patients. An additional point to consider – our CD20 data-orientated, heuristic solution to use the median value for patient stratification, although resulting in highly significant differences between high and low risk groups, still might not be optimal. Determination of the optimal cutpoint on a larger patient cohort therefore might be considered.

Subsequent questions need to be addressed – is there an interrelation between the immunological imprints of the primary and the metastatic tumors and, additionally, does the origin of infiltrating tumor cells and/or hosting tissue determine the nature of the local immune response? Prospectively, the assessment of B-cell-attributed immunological imprint and the subsequent alignment with clinicopathological parameters of both primary and matched metastatic tumors might provide a comprehensive answer. First attempt by Halama et al [58] using T-cell-attributed markers on a small patient cohort did not reveal a clear association. Thus far, the data regarding the B lymphocytes and primary CRC demonstrate that follicular structures could be indeed detected at the invasive CRC margin; the mature phenotype of isolated B cells suggested local activation and antigen-driven maturation [59], thus showing a certain level of similarity of tumor – immune cell interactions established in the colon and in the hosting liver tissue. Although speculative, we cannot exclude the possibility that tumor-instructed B cells are able to co-migrate with colon cancer cells to reside in the liver.

At the time of manuscript submission, a comprehensive study of Bindea et al was published [44] highlighting the intra-tumoral role of various immune cell sub-populations in primary colorectal cancer. Importantly, apart from T cells, the magnitude of B cells was shown to be associated with prolonged survival. In contrast, macrophages, although present at high density, had no prognostic effect in CRC patients. Thus, remarkably, there is comparable behaviour of B lymphocytes and macrophages in primary and liver metastatic colorectal cancer.

### Translational perspectives

Up-to-date recommendations from an expert panel and new consensus guidelines for management of patients with CRCLM were implementing the use of chemotherapy ± targeted agents in combination with surgery and aiming to provide a personalized approach to clinical decision making [60], [61]. Herein we introduced novel algorithm, among very few available, for quantification of immune cells within CRCLM specimens which can be further applied for comprehensive assessment of local, patient-specific immune response within diseased tissues of various etiologies. We propose the CD20-attributed immunological imprint as novel marker for CRCLM patient stratification and risk assessment. We expect that results of the current study will deliver a proposal that the herein presented patient stratification algorithm can be used as additional patient-orientated selection criterion for follow up treatment; help to direct timing of treatment administration; facilitate better stratification of patients for application of novel therapeutic approaches/drugs in clinical trials. Furthermore, patient stratification into low and high risk groups suggests tumor-promoting pathway(s)/mechanism(s) including insufficient anti-tumoral B-cell-driven responses to be evolved in a high risk group of patients. The data presented herein point out that activation of B cells via various routes including the therapeutic agonistic antibodies (by way of example, agonistic CD40 antibodies; reviewed in [62]) might be considered for proposal as novel treatment strategies for CRCLM patients.

## Supporting Information

Figure S1 Algorithm for automatic detection and quantification of infiltrating immune cells. Quantitative analysis using the TissueQuest/HistoQuest software was applied to determine amounts of CD45-positive, CD20-positive, and CD68-positive cells within three locations of interest. For CD45 (*a*, red channel for CD45 and blue channel for nuclei/DAPI) and CD20 (*c*, brown staining for CD20 and blue for nuclei/haematoxilin) as molecules expressed on the cell surface, the calculations are based on the recognition of each nucleus (size and staining intensity as major parameters) followed by the analysis of specific staining. Various cell types within the specimen characterized by different nuclear size and staining intensities (including among others hepatocytes, colon cancer epithelial cells, and immune cells) were simultaneously recognized and examined through the region of interest. The percentage of positively stained cells is calculated by the software relative to the total number of cells within the examined region of interest based on the automatic recognition of individual cells: *red* circles indicate CD45-positive (*b*) and CD20-positive (*d*) cells, while negative cells are marked in *green*. A different strategy was found to be optimal to evaluate the staining for CD68 since subcellular localization of the CD68 molecule is predominantly attributed to lysosomal/endosomal membranes within the cytoplasmic area. Thus, nucleus as cell identification marker used by the software is often covered by the CD68-specific staining; therefore, for CD68 (*e*), the total area of positively stained cells relative to the total area of the examined region of interest is calculated in percentage (*f*, for better visualization, areas of individual positive cells are marked by various colors).
(TIF)

Figure S2 Comparative analysis of CD45 data sets for specimens from panel I versus panel II. Boxplots of CD45 values of panel I (n = 13) and panel II (n = 19) specimens are shown for three regions of interest. The boxplot represents the distribution of values; the line across the box represents the median; the box stretches from the lower hinge (the 25th percentile) to the upper hinge (the 75th percentile); t test; ns, not significant.
(TIF)

Figure S3 Comparative analysis of CD20 data sets for specimens from panel I versus panel II. Boxplots of CD20 values of panel I (n = 11) and panel II (n = 51) specimens are shown for three regions of interest. The boxplot represents the distribution of values; the line across the box represents the median; the box stretches from the lower hinge (the 25th percentile) to the upper hinge (the 75th percentile); t test; ns, not significant.
(TIF)

Figure S4 Highly organized ectopic follicular structures are characterized by the presence of embedded bile ducts. (*a*) HE staining and (*b*) cytokeratin 19 staining (merged image, red color, cytokeratin 19; blue color, DAPI) of ectopic follicles at the tumor – liver border with multiple bile ducts. Scale bar: 50 µm.
(TIF)

Figure S5 Comparison of panel I and panel II regarding the ectopic follicle score. Analysis was done using chi-square trend test and was based on the scoring system used for characterization of the presence of follicular structures (no, low, high); panel I, n = 14; panel II, n = 51. Shown is percentage of patients characterized by “no”, “low” or “high” ectopic follicle score; the total number of patients per panel is set to 100%.
(TIF)

Figure S6 Comparative analysis of CD68 data sets for specimens from panel I versus panel II. Boxplots of CD68 values of panel I (n = 12) and panel II (n = 51) specimens are shown for three regions of interest. The boxplot represents the distribution of values; the line across the box represents the median; the box stretches from the lower hinge (the 25th percentile) to the upper hinge (the 75th percentile); t test; ns, not significant.
(TIF)

Table S1 Univariate and multivariate Cox regression analyses of CD45 staining-derived data sets at three regions of interest for RFS and OS.
(TIF)

Table S2 Testing of staining-derived data sets for interaction with the panel as variable for RFS and OS.
(TIF)

Table S3 Comparison of staining-derived data sets and the ectopic follicle score between the two chemotherapy groups within panel II.
(PDF)

Table S4 Univariate and multivariate Cox regression analyses of CD20 staining-derived data sets at three regions of interest for RFS and OS.
(TIF)

Table S5 Univariate and multivariate Cox regression analyses of the ordinal variable “ectopic follicles” for RFS and OS.
(TIF)

Table S6 Univariate and multivariate Cox regression analyses of CD68 staining-derived data sets at three regions of interest for RFS and OS.
(TIF)

Table S7 Correlation matrix for staining-derived variables.
(TIF)

Table S8 Univariate and multivariate Cox regression analyses of clinical variables for RFS and OS.
(TIF)
