# Supplementary figures and images for: B Cells and Ectopic Follicular Structures: Novel Players in Anti-Tumor Programming with Prognostic Power for Patients with Metastatic Colorectal Cancer
Source: PLoS One. 2014 Jun 6;9(6):e99008. doi: 10.1371/journal.pone.0099008 (PMC4048213; doi:10.1371/journal.pone.0099008)

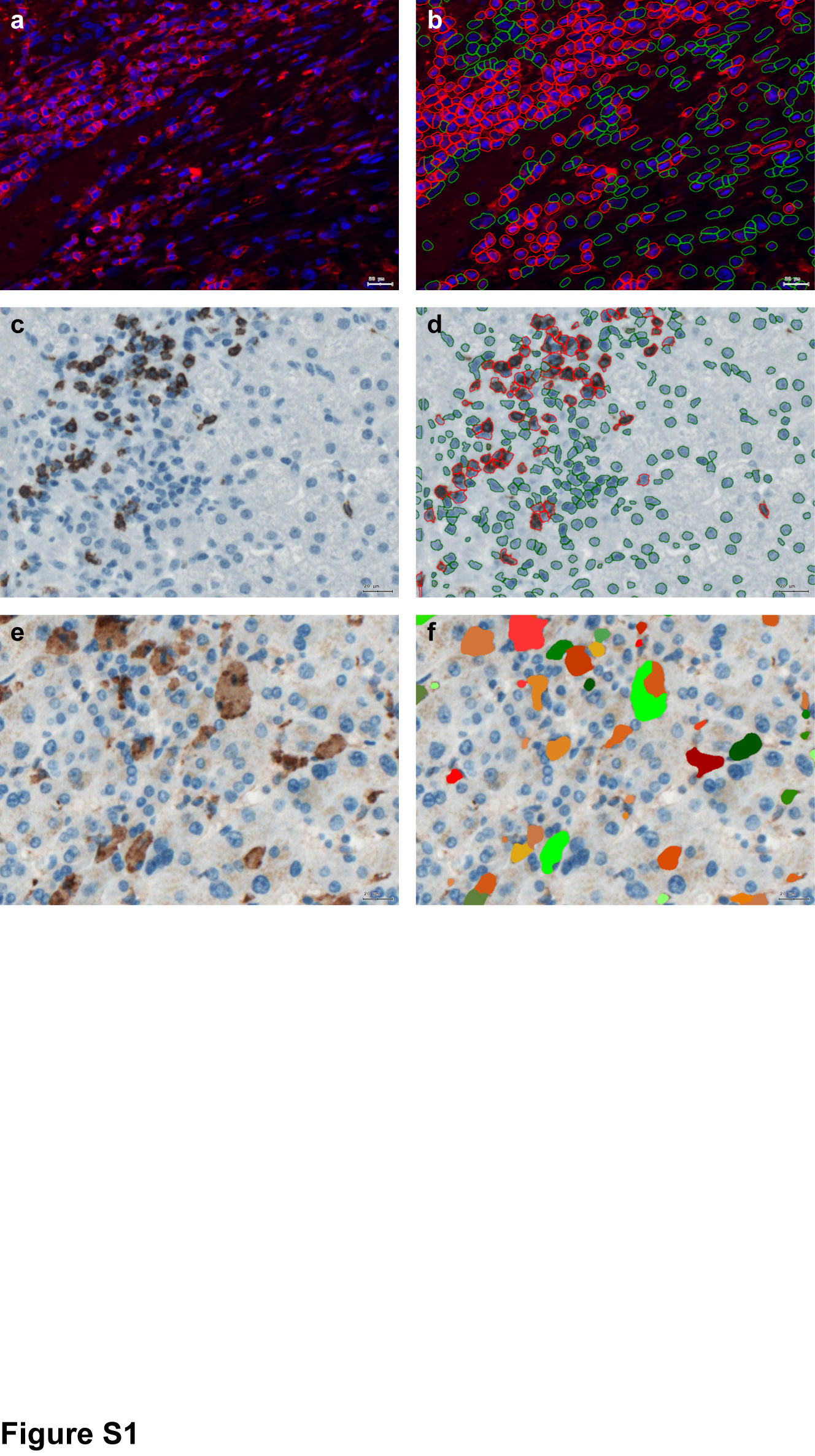

Supplement: Figure S1 — Algorithm for automatic detection and quantification of infiltrating immune cells. Quantitative analysis using the TissueQuest/HistoQuest software was applied to determine amounts of CD45-positive, CD20-positive, and CD68-positive cells within three locations of interest. For CD45 (a, red channel for CD45 and blue channel for nuclei/DAPI) and CD20 (c, brown staining for CD20 and blue for nuclei/haematoxilin) as molecules expressed on the cell surface, the calculations are based on the recognition of each nucleus (size and staining intensity as major parameters) followed by the analysis of specific staining. Various cell types within the specimen characterized by different nuclear size and staining intensities (including among others hepatocytes, colon cancer epithelial cells, and immune cells) were simultaneously recognized and examined through the region of interest. The percentage of positively stained cells is calculated by the software relative to the total number of cells within the examined region of interest based on the automatic recognition of individual cells: red circles indicate CD45-positive (b) and CD20-positive (d) cells, while negative cells are marked in green. A different strategy was found to be optimal to evaluate the staining for CD68 since subcellular localization of the CD68 molecule is predominantly attributed to lysosomal/endosomal membranes within the cytoplasmic area. Thus, nucleus as cell identification marker used by the software is often covered by the CD68-specific staining; therefore, for CD68 (e), the total area of positively stained cells relative to the total area of the examined region of interest is calculated in percentage (f, for better visualization, areas of individual positive cells are marked by various colors). (TIF) [file pone.0099008.s001.tif]

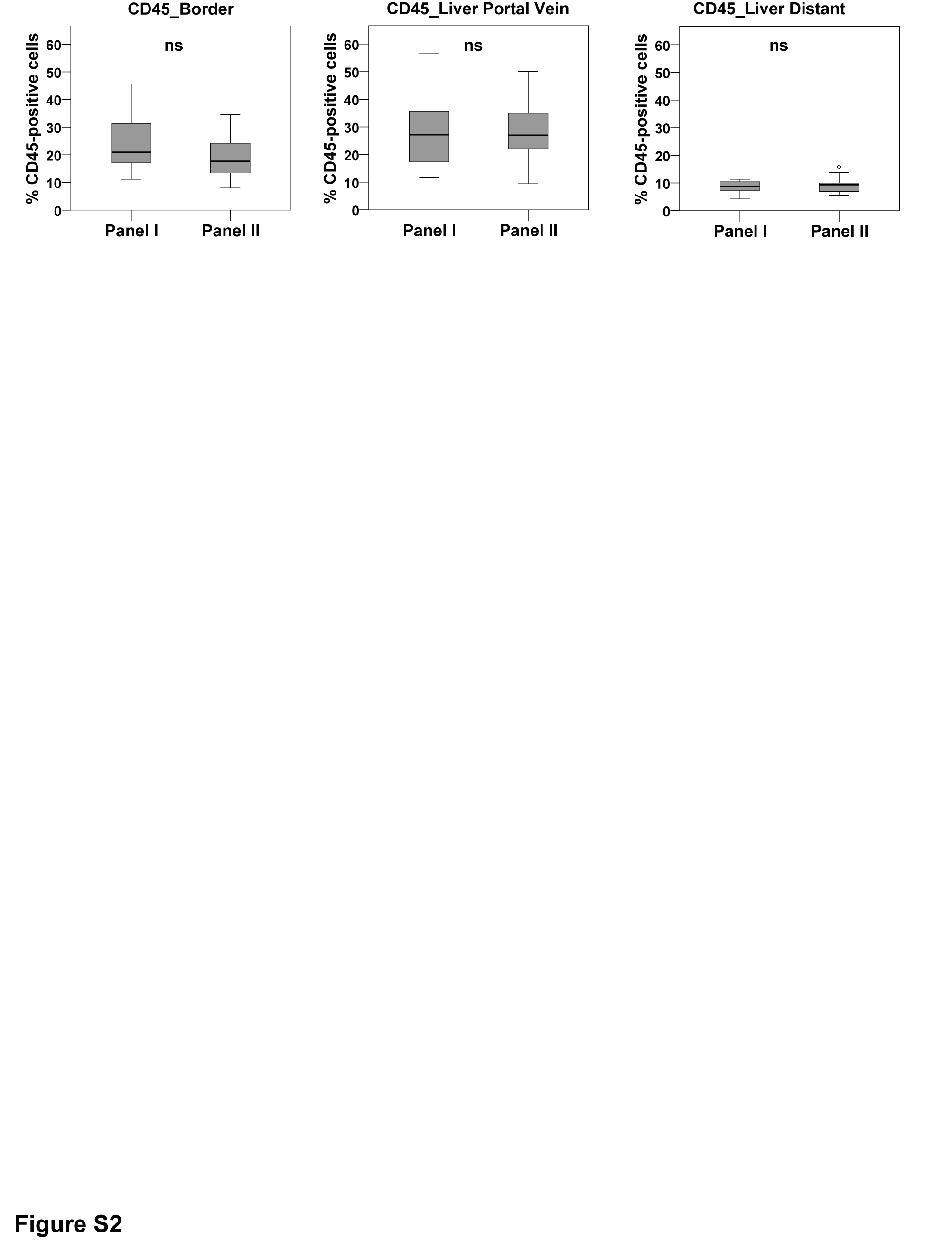

Supplement: Figure S2 — Comparative analysis of CD45 data sets for specimens from panel I versus panel II. Boxplots of CD45 values of panel I (n = 13) and panel II (n = 19) specimens are shown for three regions of interest. The boxplot represents the distribution of values; the line across the box represents the median; the box stretches from the lower hinge (the 25th percentile) to the upper hinge (the 75th percentile); t test; ns, not significant. (TIF) [file pone.0099008.s002.tif]

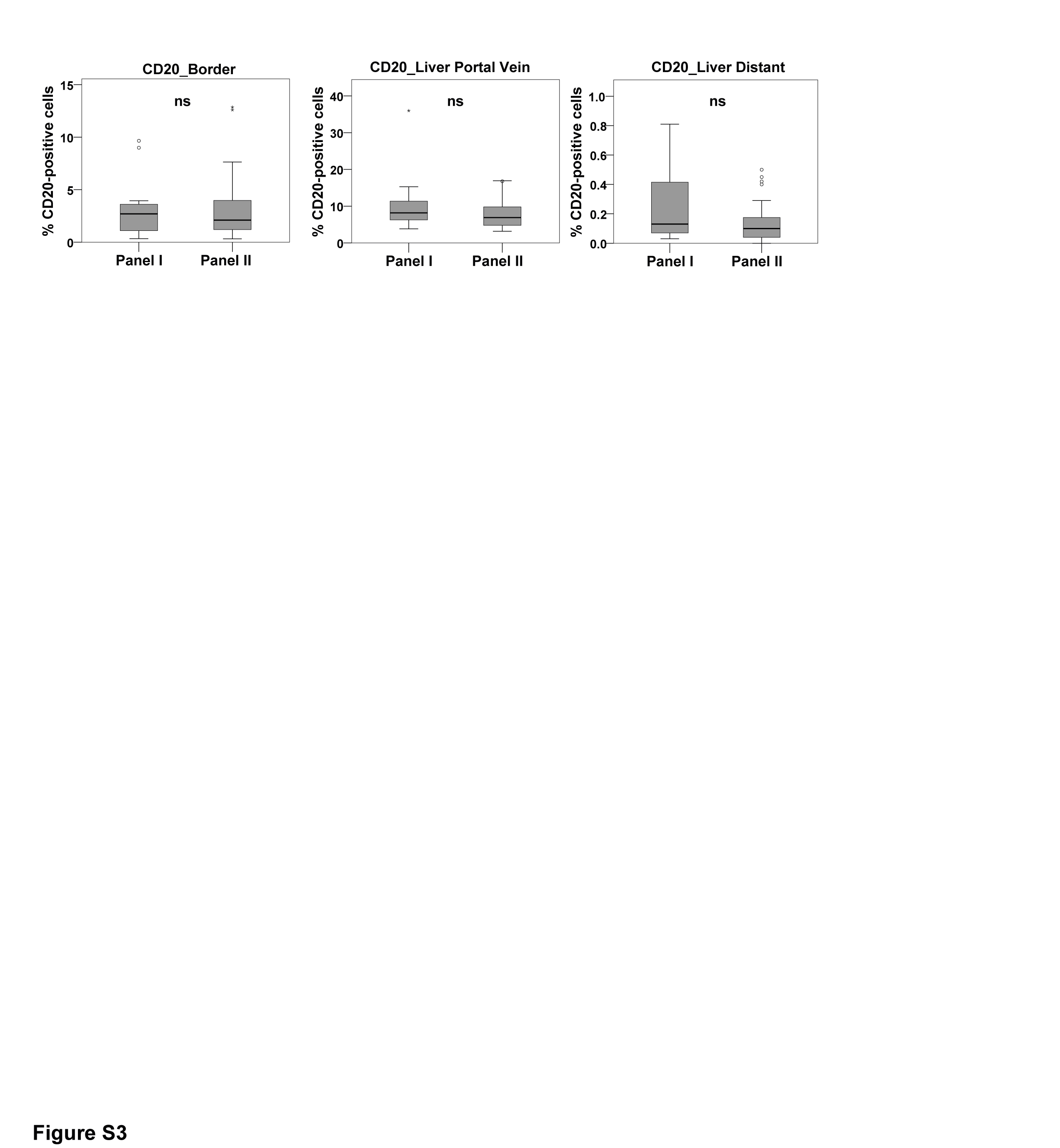

Supplement: Figure S3 — Comparative analysis of CD20 data sets for specimens from panel I versus panel II. Boxplots of CD20 values of panel I (n = 11) and panel II (n = 51) specimens are shown for three regions of interest. The boxplot represents the distribution of values; the line across the box represents the median; the box stretches from the lower hinge (the 25th percentile) to the upper hinge (the 75th percentile); t test; ns, not significant. (TIF) [file pone.0099008.s003.tif]

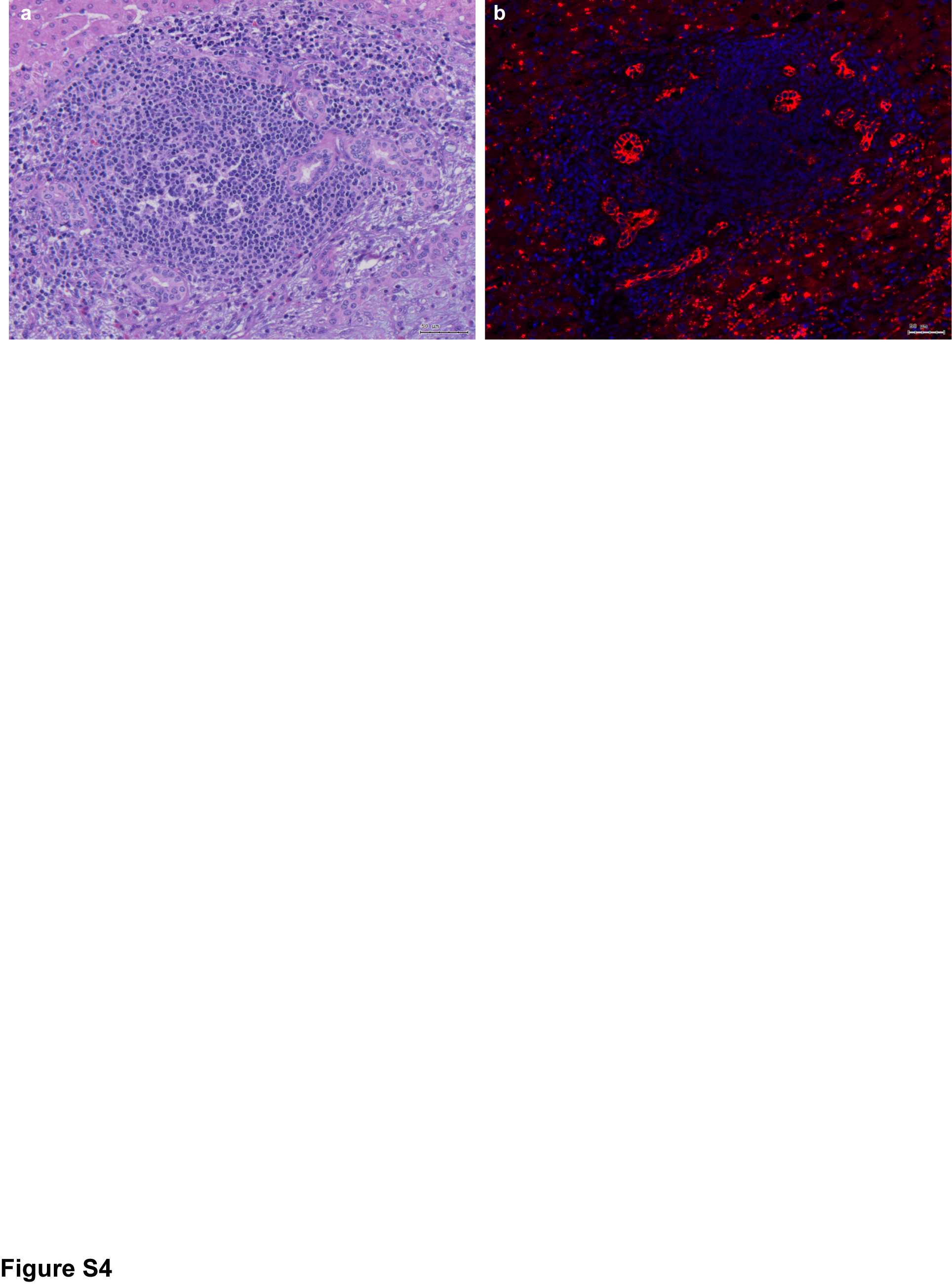

Supplement: Figure S4 — Highly organized ectopic follicular structures are characterized by the presence of embedded bile ducts. (a) HE staining and (b) cytokeratin 19 staining (merged image, red color, cytokeratin 19; blue color, DAPI) of ectopic follicles at the tumor – liver border with multiple bile ducts. Scale bar: 50 µm. (TIF) [file pone.0099008.s004.tif]

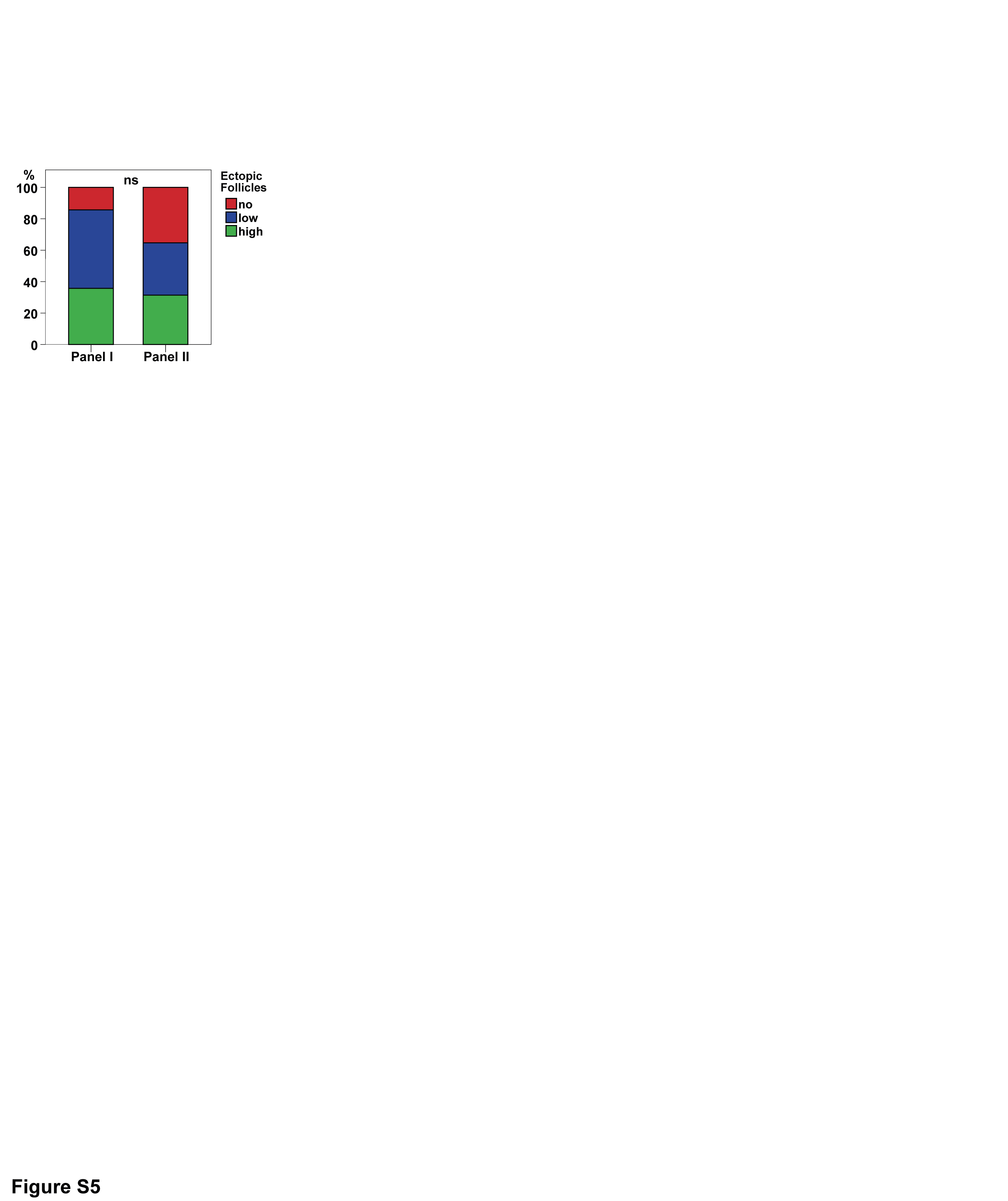

Supplement: Figure S5 — Comparison of panel I and panel II regarding the ectopic follicle score. Analysis was done using chi-square trend test and was based on the scoring system used for characterization of the presence of follicular structures (no, low, high); panel I, n = 14; panel II, n = 51. Shown is percentage of patients characterized by “no”, “low” or “high” ectopic follicle score; the total number of patients per panel is set to 100%. (TIF) [file pone.0099008.s005.tif]

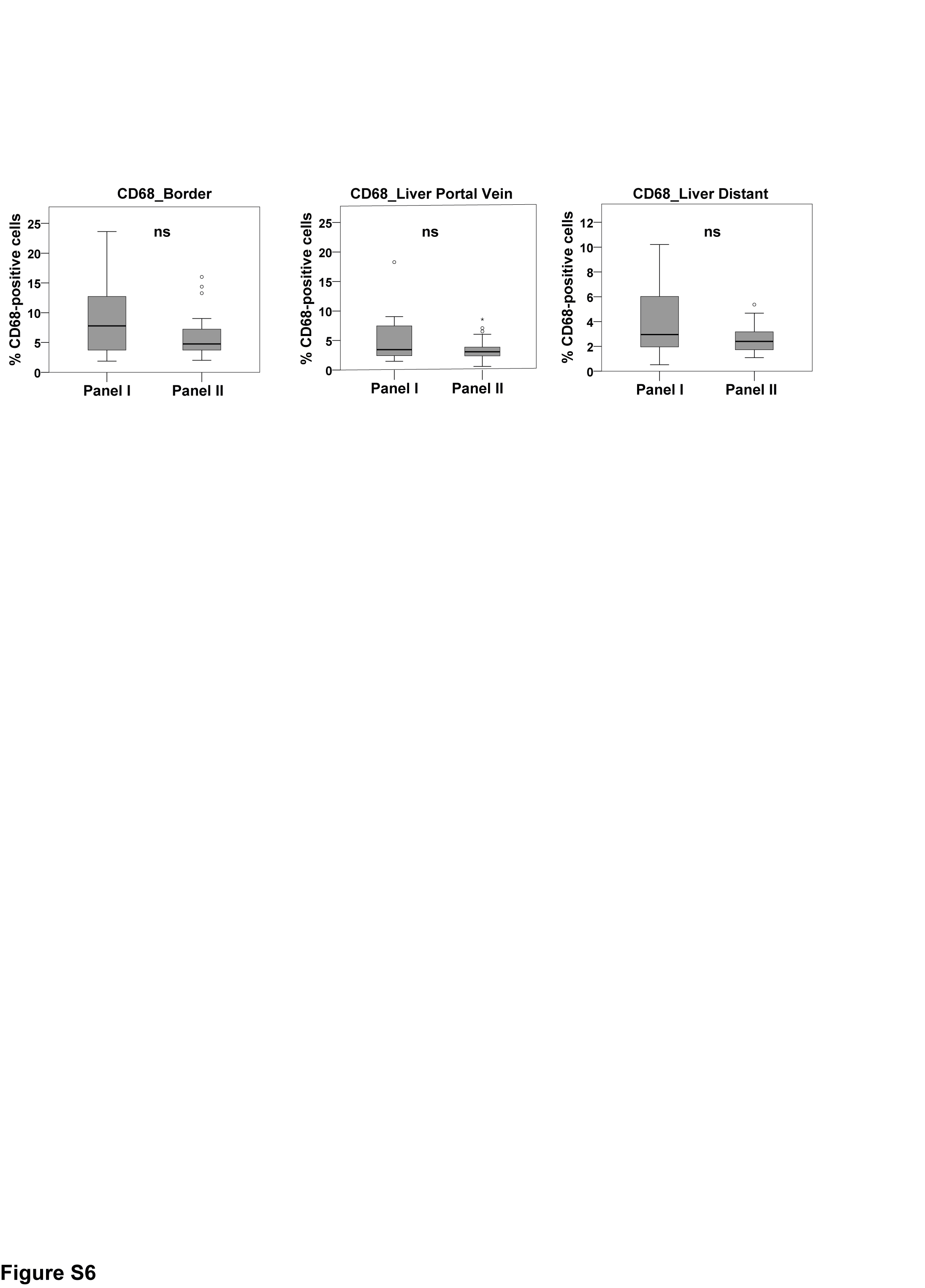

Supplement: Figure S6 — Comparative analysis of CD68 data sets for specimens from panel I versus panel II. Boxplots of CD68 values of panel I (n = 12) and panel II (n = 51) specimens are shown for three regions of interest. The boxplot represents the distribution of values; the line across the box represents the median; the box stretches from the lower hinge (the 25th percentile) to the upper hinge (the 75th percentile); t test; ns, not significant. (TIF) [file pone.0099008.s006.tif]

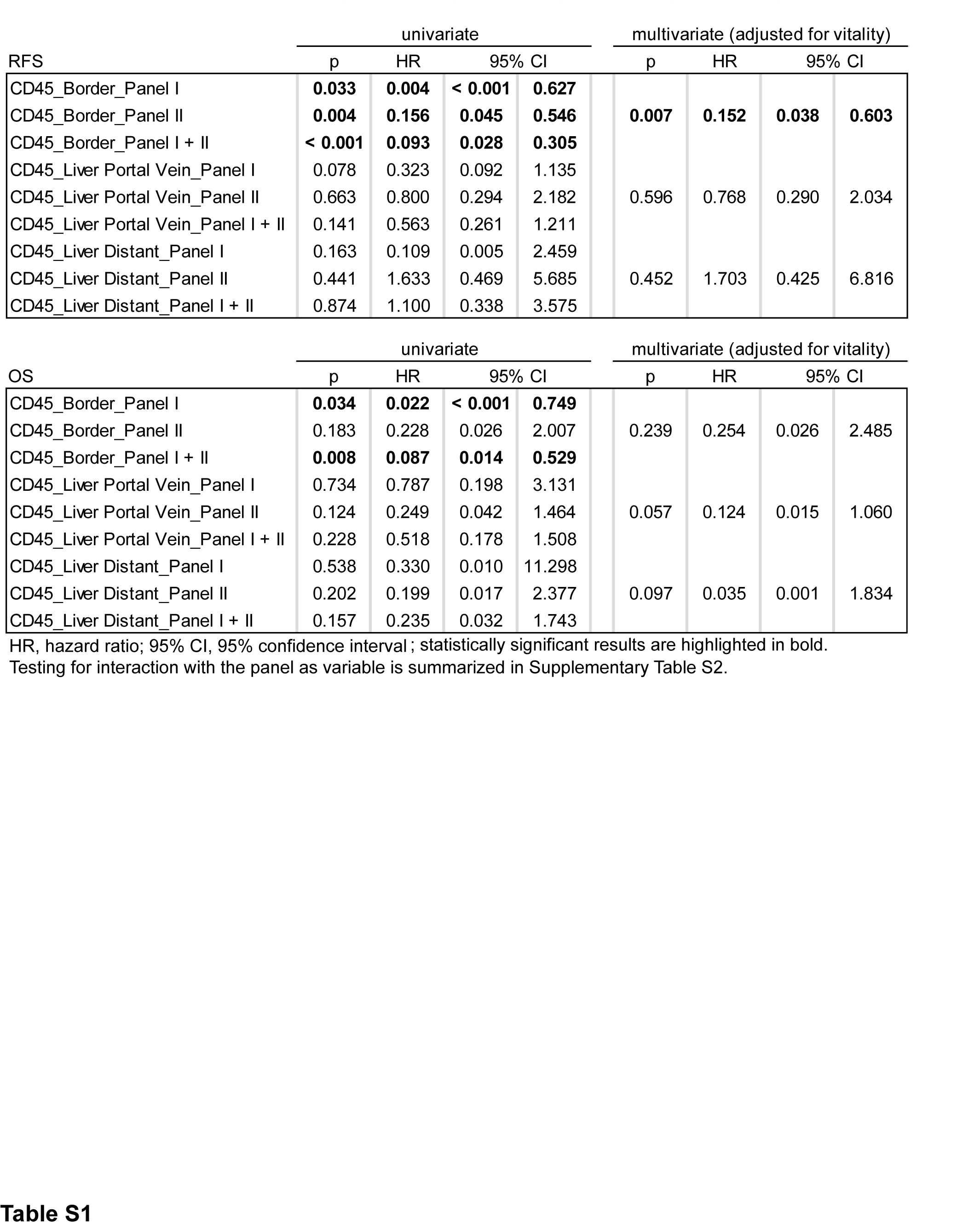

Supplement: Table S1 — Univariate and multivariate Cox regression analyses of CD45 staining-derived data sets at three regions of interest for RFS and OS. (TIF) [file pone.0099008.s007.tif]

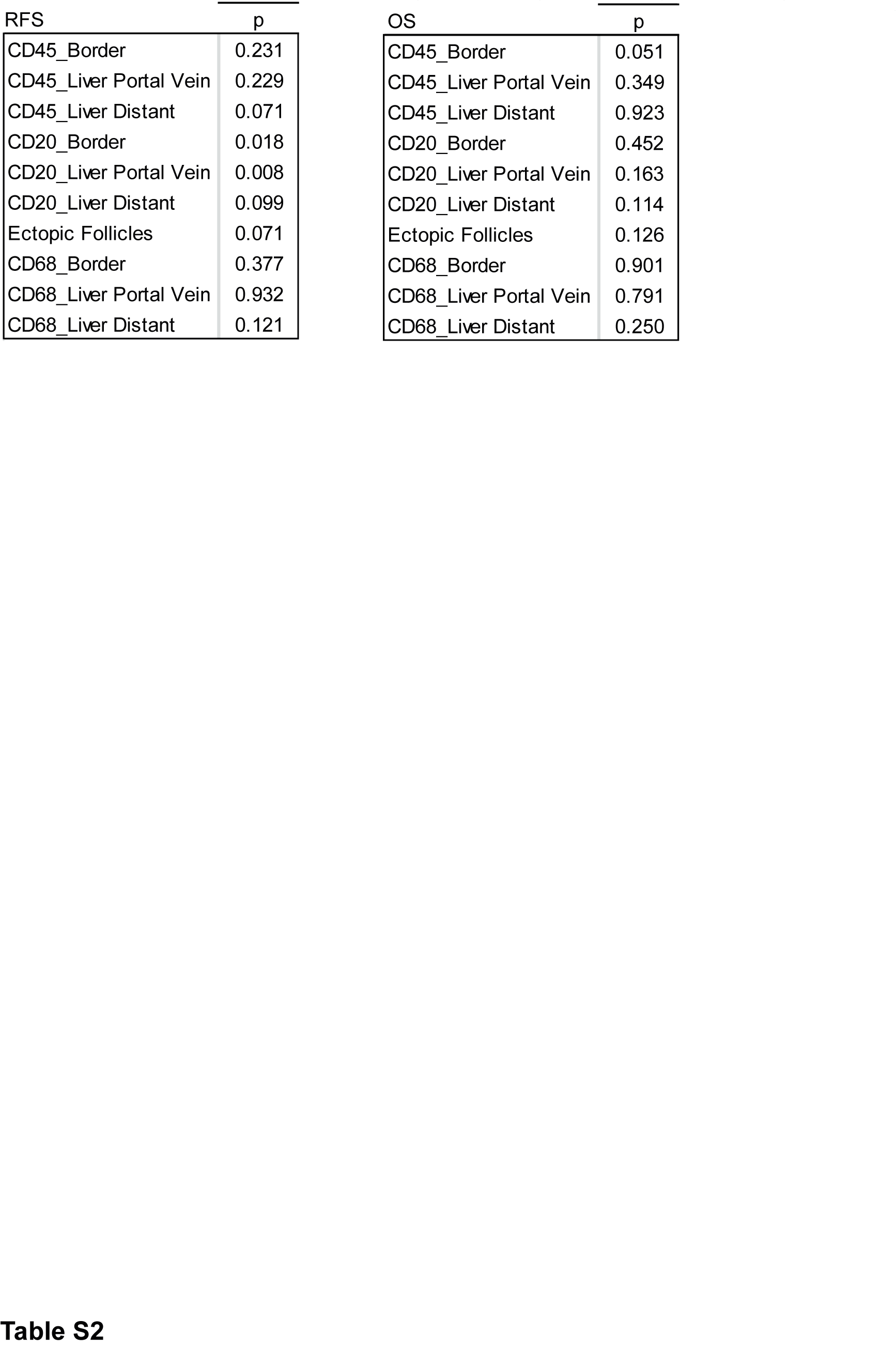

Supplement: Table S2 — Testing of staining-derived data sets for interaction with the panel as variable for RFS and OS. (TIF) [file pone.0099008.s008.tif]

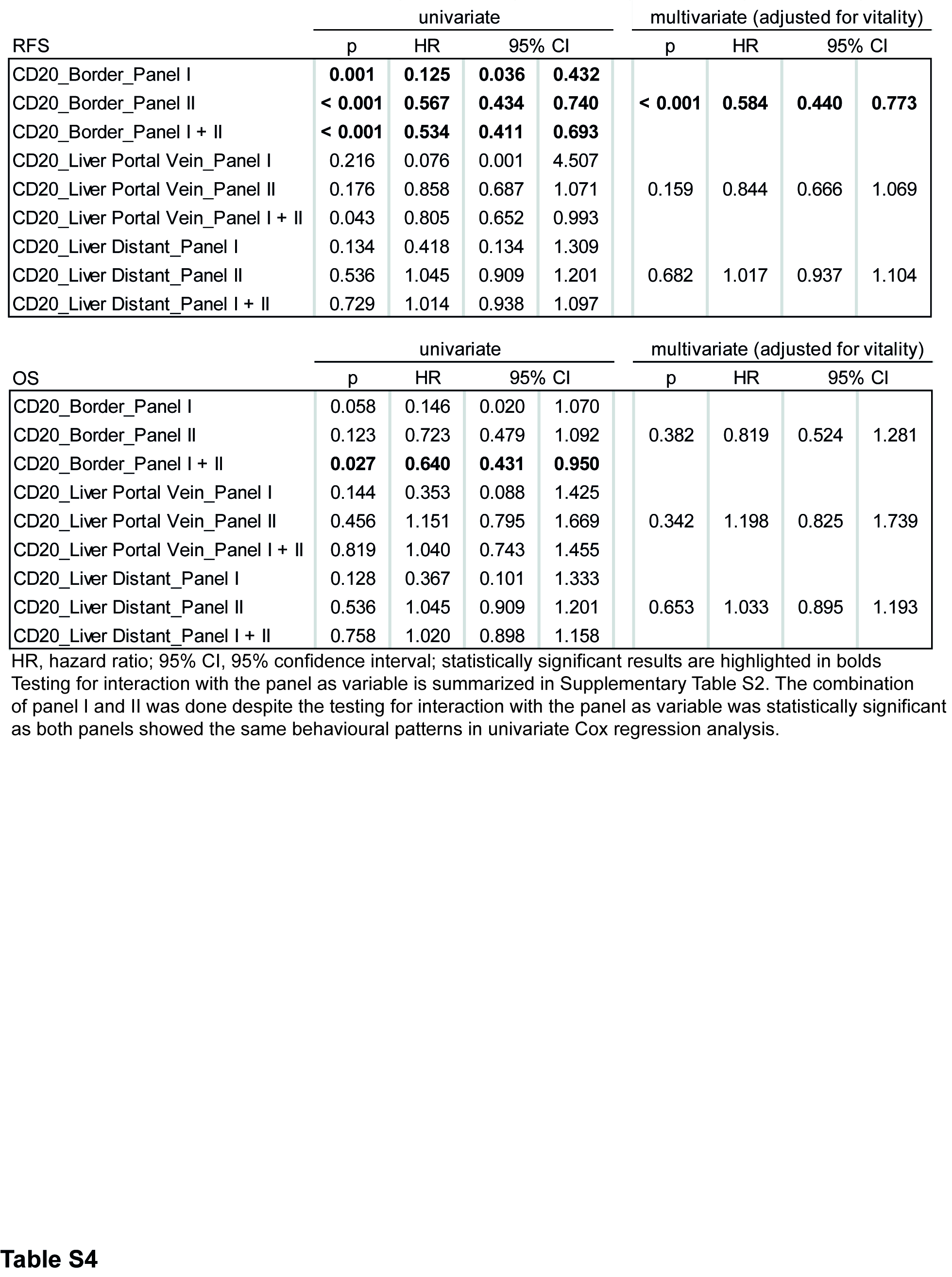

Supplement: Table S4 — Univariate and multivariate Cox regression analyses of CD20 staining-derived data sets at three regions of interest for RFS and OS. (TIF) [file pone.0099008.s010.tif]

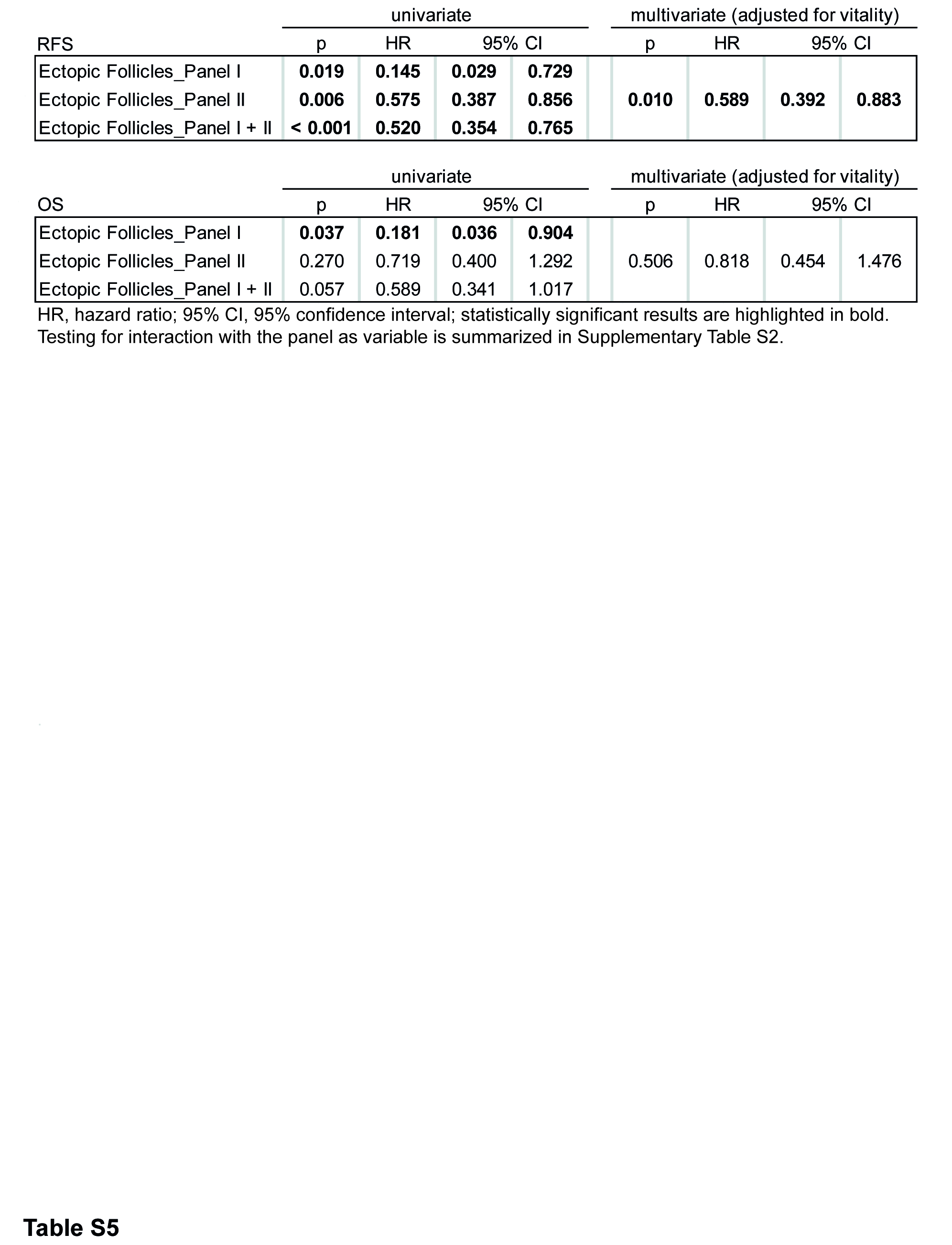

Supplement: Table S5 — Univariate and multivariate Cox regression analyses of the ordinal variable “ectopic follicles” for RFS and OS. (TIF) [file pone.0099008.s011.tif]

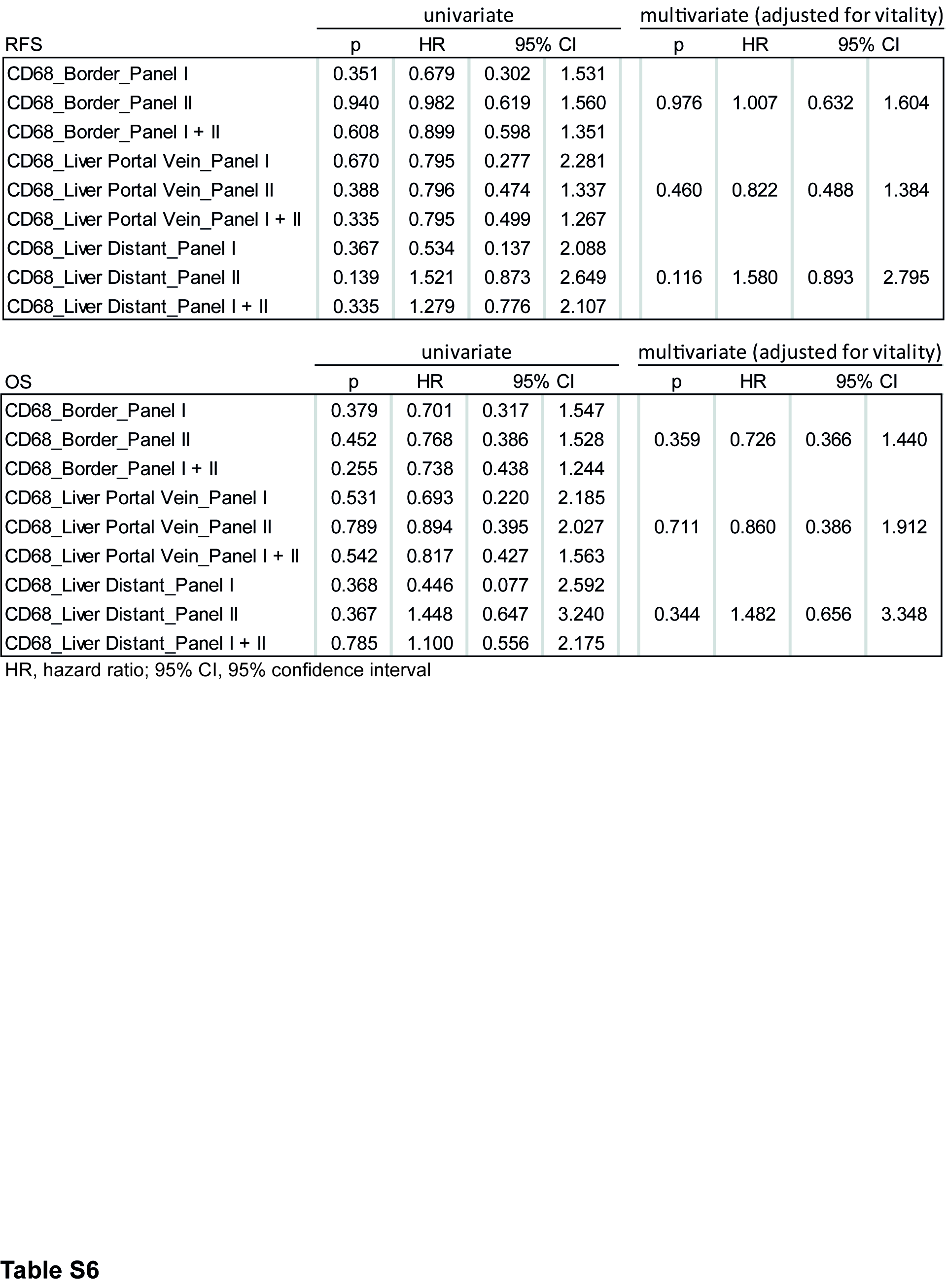

Supplement: Table S6 — Univariate and multivariate Cox regression analyses of CD68 staining-derived data sets at three regions of interest for RFS and OS. (TIF) [file pone.0099008.s012.tif]

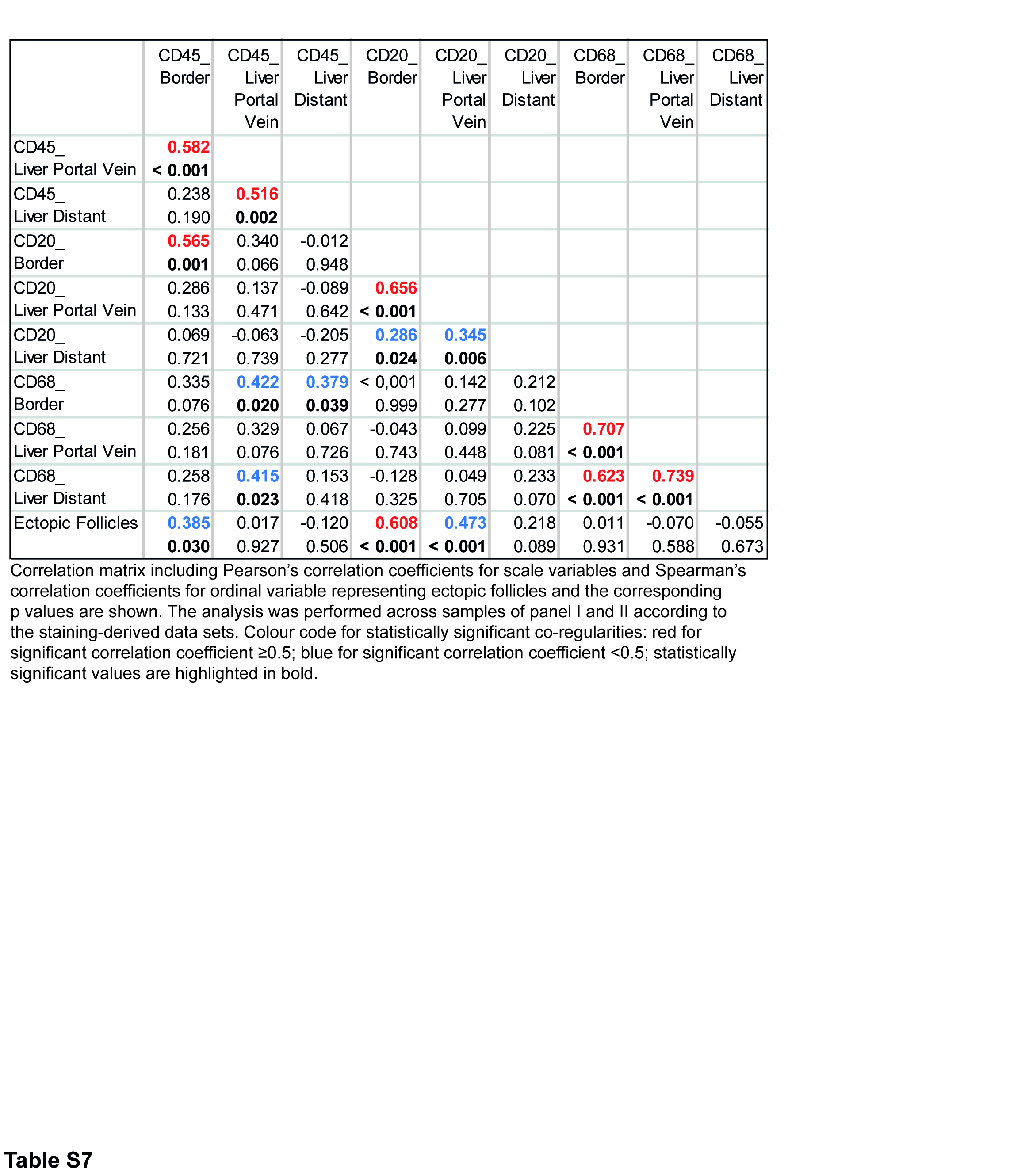

Supplement: Table S7 — Correlation matrix for staining-derived variables. (TIF) [file pone.0099008.s013.tif]

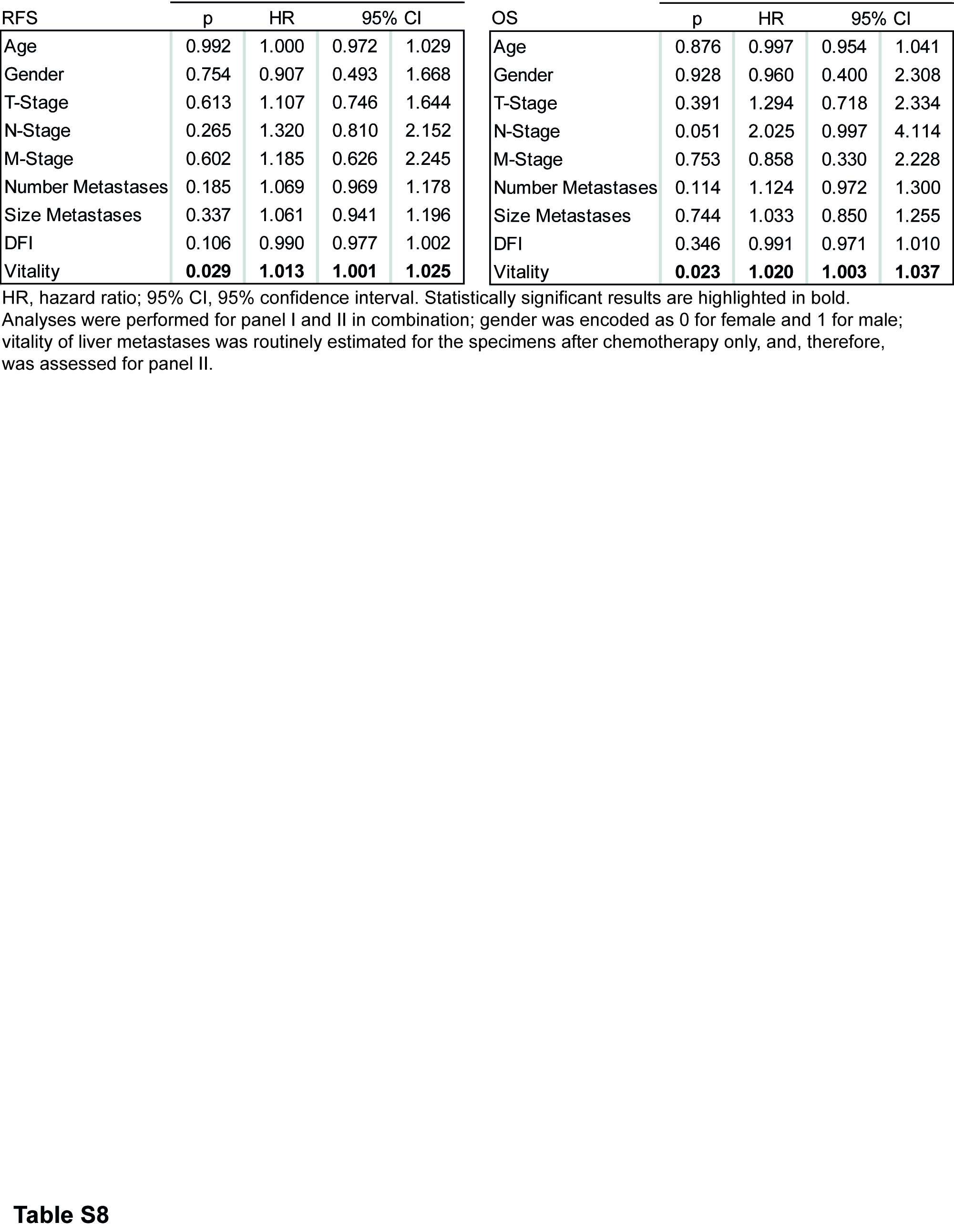

Supplement: Table S8 — Univariate and multivariate Cox regression analyses of clinical variables for RFS and OS. (TIF) [file pone.0099008.s014.tif]
