# Supplementary material for: B Cells and Ectopic Follicular Structures: Novel Players in Anti-Tumor Programming with Prognostic Power for Patients with Metastatic Colorectal Cancer
Source: PLoS One. 2014 Jun 6;9(6):e99008. doi: 10.1371/journal.pone.0099008 (PMC4048213; doi:10.1371/journal.pone.0099008)
Supplement: Table S3 — Comparison of staining-derived data sets and the ectopic follicle score between the two chemotherapy groups within panel II. (PDF) [file pone.0099008.s009.pdf]

	Oxaliplatin<br>median (min– max)	Irinotecan<br>median (min–max)
CD45_Border_Panel II	4.2 (3.0 – 5.1)	3.8*
CD45_Liver Portal Vein_Panel II	4.7 (3.2 – 5.7)	5.1*
CD45_Liver Distant_Panel II	3.2 (2.5 – 4.0)	3.6*
CD20_Border_Panel II	1.1 (-1.6 – 3.7)	1.6 (-1.6 – 2.1)
CD20_Liver Portal Vein_Panel II	2.0 (-3.6 – 4.1)	2.7 (-0.3 – 3.5)
CD20_Liver Distant_Panel II	-3.5 (-13.3 – -1.0)	-2.3 (-4.3 – -1.3)
CD68_Border_Panel II	2.3 (1.0 – 4.0)	2.1 (1.6 – 3.9)
CD68_Liver Portal Vein_Panel II	1.7 (0.1 – 3.1)	1.4 (-0.7 – 1.7)
CD68_Liver Distant_Panel II	1.3 (0.1 – 2.2)	0.7 (0.3 – 2.4)
Ectopic Follicles	no – 16 (34.8%)<br>low – 15 (32.6%)<br>high – 15 (32.6%)	no – 2 (40.0%)<br>low – 2 (40.0%)<br>high – 1 (20.0%)

Log2 transformed values for continuous variables are shown. Continuous data were described with median, minimum and maximum; categorical data were described by absolute and relative frequencies. *CD45 data sets include one measurement for irinotecan and 18 values for oxaliplatin; thus, the single measurement value for irinotecan is shown. Due to the small number of observations in the irinotecan group, statistical tests have a small power to detect differences and a pure description of the data is more appropriate.

Table S3
